# A scientometric analysis of research related to the ‘oral-placental axis’ hypothesis: current status, hotspots, and future directions

**DOI:** 10.3389/fcimb.2026.1864022

**Published:** 2026-06-30

**Authors:** Beiying Zhang, Zhouhui Yun, Yi Yuan, Lijie Li, Zhihui Xiong

**Affiliations:** 1 Stomatology Hospital, School of Stomatology, Zhejiang University School of Medicine, Hangzhou, China; 2 Obstetrical Department, Zhejiang Hospital, Hangzhou, China

**Keywords:** adverse pregnancy outcomes, conceptual evolution, knowledge graph, molecular mechanisms, oral-placental axis, periodontal diseases, research trends, scientometrics

## Abstract

Oral diseases and placental disorders are closely associated with adverse pregnancy outcomes, and accumulating evidence supports their crosstalk that constitutes the “oral−placental axis”. This study integrated scientometric and bioinformatic approaches to systematically analyze global research trends, collaboration networks, research hotspots, and core molecular−microbial mechanisms of the oral−placental axis covering the period from 2016 to 2025. A total of 196 eligible publications were retrieved from the Web of Science Core Collection, Scopus, and PubMed. Bibliometric visualization was performed using VOSviewer, CiteSpace, and R−bibliometrix, and bioinformatic analysis was conducted to identify shared genes, signaling pathways, and microbial links between oral and placental diseases. The results revealed an annual publication growth rate of 5.03%, with the United States, China, and Australia as major contributing countries, the University of Queensland as the leading institution, and Gomez−Arango Luisa F. and Nitert Marloes Dekker as the most influential authors. Core keywords included preterm birth, periodontal diseases, gestational diabetes mellitus, and oral microbiome, reflecting a research shift from phenotypic association to mechanistic exploration such as microbial vertical transmission and inflammatory signaling. Mechanistic analyses identified shared hub genes (e.g., KRT19, ADAMDEC1, AQP9, SPAG4, PLAT) and key pathways, predominantly primary immunodeficiency and complement and coagulation cascades. Pathogenic bacteria including *Fusobacterium nucleatum* and *Porphyromonas gingivalis* mediated adverse pregnancy outcomes via hematogenous spread and placental barrier disruption. This study established a bidirectional regulatory model of the oral−placental axis involving shared risks, microbial transmission, and systemic inflammation, providing a theoretical basis for preconception oral intervention and precise prevention during pregnancy, and supporting the integration of oral care into routine perinatal management.

## 1 Introduction

Since its explicit formulation in the early 21st century, the oral-placental axis hypothesis has emerged as a central research paradigm at the intersection of stomatology and obstetrics and gynecology (Bobetsis et al., 2020). This hypothesis posits that local infections originating from the oral cavity—particularly periodontal pathogens and the inflammatory mediators they trigger—may breach local barriers and reach the placental microenvironment via the bloodstream or other routes, thereby contributing to adverse pregnancy outcomes such as preterm birth, low birth weight, and preeclampsia (Nannan et al., 2022; AlSharief and Alabdurubalnabi, 2023). Over the past decade, numerous basic, clinical, and epidemiological studies have been conducted around this hypothesis, yielding a body of evidence accompanied by ongoing debates. A large number of observational studies have confirmed a statistical association between periodontal disease and adverse pregnancy outcomes (Pockpa et al., 2021). For instance, a large retrospective cohort study covering more than 740,000 pregnancy records found that mothers with periodontal disease had significantly elevated risks of any adverse pregnancy outcome, low birth weight, preterm birth, and spontaneous abortion (Choi et al., 2021). Regional studies conducted in Saudi Arabia, Nepal, and other locations also support periodontal disease as an independent risk factor for preterm birth and low birth weight (Alrumayh et al., 2021; Sharma et al., 2024). Collectively, these studies point to a consistent conclusion: periodontal disease, a prevalent chronic oral infection, may pose a substantial threat to pregnancy.

Despite the growing body of epidemiological evidence, the underlying biological mechanisms and the certainty of causality remain controversial. Two major pathways—direct and indirect—are currently widely recognized (Figuero et al., 2020). The direct pathway refers to the hematogenous dissemination of oral pathogens to the placenta via bacteremia, inducing local infection and inflammation that disrupt the stability of the fetoplacental unit (Guan et al., 2023). Periodontal pathogens, including Porphyromonas gingivalis and Fusobacterium nucleatum, have been detected in the cord blood and placental tissues of preterm infants, providing strong evidence for direct transmission (Savitha et al., 2022; Hakobyan et al., 2024). The indirect pathway involves the systemic inflammatory response triggered by periodontal infection. The release of pro-inflammatory cytokines (e.g., interleukin-6) and prostaglandins remotely impairs placental function, predisposing to preterm birth or preeclampsia (Jagannathan et al., 2021; Wen et al., 2023). For example, it has been hypothesized that CD14+CD16+ monocytes may serve as a causal link between periodontitis and adverse pregnancy outcomes (Jagannathan et al., 2021). Furthermore, anticardiolipin antibodies induced by periodontal pathogens have been shown to interfere with trophoblast function via molecular mimicry, leading to pregnancy failure (Schenkein and Thomas, 2020). Extracellular vesicles released by macrophages infected with Porphyromonas gingivalis, as well as bacterial outer membrane vesicles, can translocate to the fetoplacental unit, impair placental angiogenesis and function, and consequently affect fetal development (Lara et al., 2023; Tanai et al., 2025).

Scientometric approaches provide powerful tools for objectively and quantitatively evaluating the overall development, evolving knowledge structure, and cutting-edge trends in this field (Zhao et al., 2025), while bioinformatics analysis enables the dissection of regulatory mechanisms underlying the oral-placental axis at the molecular level, thereby providing empirical support for this hypothesis. This study employs an integrated design combining scientometric and bioinformatics analyses to systematically investigate the association between oral health and pregnancy outcomes from macroscopic trends and microscopic mechanisms. First, using scientometric methods, we quantitatively and visually analyze extensive bibliographic data over the past decade to reveal its research trajectory, knowledge structure, and current frontiers (Wu et al., 2024), and elucidate macro-level factors contributing to inconsistent conclusions between observational studies and RCTs (Xu et al., 2025). Second, we use bioinformatics analysis to explore shared molecular pathways and regulatory networks between oral diseases and placenta-related disorders leveraging public high-throughput datasets, providing molecular evidence for the hypothesis. Finally, integrating qualitative analysis, we interpret findings from bibliometrics and omics to summarize core consensus, clarify controversies, and propose future directions. This combination enhances the scientific validity and reliability of the conclusions. Notably, although numerous observational studies support an association, interventional RCTs of periodontal treatment yield inconsistent results, sparking discussions regarding causality (Acharya et al., 2026). Many high-quality RCTs demonstrate that second-trimester non-surgical periodontal treatment does not significantly improve pregnancy outcomes, potentially attributable to the timing of the intervention being insufficient to prevent pathogen colonization and placental damage (Caneiro-Queija et al., 2019a). Heterogeneity in study design also contributes to discrepant findings. Future research requires more rigorously designed studies to verify this relationship. Furthermore, with a deeper understanding of oral dysbiosis, research focus has expanded to the oral microbiome-placental axis, exploring how pregnancy-related hormonal and immunological changes induce oral dysbiosis that regulates pregnancy outcomes via the placental microbiome or systemic status (Ye and Kapila, 2021).

## 2 Methods

Scientometrics, as a bibliometric application in scientific research, facilitates the systematic collation of massive literature data and reveals the evolutionary trends and emerging directions of specific disciplines (Ye and Kapila, 2021). In the field of oral-placental axis research, scientometrics not only assists researchers in accurately identifying the landmark research achievements in this field but also elucidates key research hotspots and identifies currently underexplored gaps within studies correlating oral health status, placental physiological function, and pregnancy outcomes (Yang et al., 2024).

### 2.1 Data sources

For this study, a literature search was conducted in three core biomedical databases: the Web of Science Core Collection (WOSCC), Scopus, and PubMed, covering the period from January 1, 2016, to December 31, 2025. Keywords were selected based on broad definitions and relevant terminology in the field, including standard medical terminology, Medical Subject Headings (MeSH), and synonyms related to the oral cavity and placenta. The search strategy combined the following Boolean logic: TS = (“oral cavity” OR “oral region” OR “dental” OR “oromandibular system” OR “buccal region” OR “stomatognathic system”) AND TS = (“placenta” OR “placental” OR “afterbirth” OR “fetal membrane” OR “placental tissue”) AND TS = (“periodontal disease” OR “gingivitis” OR “pregnancy outcome” OR “preterm birth” OR “preeclampsia”). To ensure scientific rigor and consistency, specific exclusion criteria were applied: animal studies, non-original research, non-review articles, and non-English publications were excluded. Consequently, only original research papers and reviews published in English were retained for subsequent analysis. Using this search protocol, the initial search results were as follows: 808 records from the Web of Science Core Collection, 1080 records from Scopus, and 162 records from PubMed, which together constituted the initial dataset for subsequent analysis (**Supplementary Table 1** and **Supplementary Figure 1**). This search strategy ensured the objectivity and reproducibility of the research process and provided systematic coverage of research on the oral-placental axis.

### 2.2 Bibliometric analysis

To systematically explore research trends and molecular regulatory mechanisms of the oral-placental axis, we adopted an integrated strategy combining scientometric and bioinformatic analyses. Bibliometric analysis was performed using CiteSpace 6.2.R4, VOSviewer 1.6.20, Pajek, and the R package bibliometrix (4.2.2). CiteSpace was applied for keyword clustering and identification of research frontiers, while VOSviewer was used for network visualization, Pajek for analyzing the topology of collaboration networks, and bibliometrix in conjunction with ggplot2 for data preprocessing and quantitative statistical analysis. Uniform analytical parameters were set to ensure methodological rigor: default settings for bibliometrix, full counting mode for VOSviewer, a 2016–2025 time span with 1-year slices for CiteSpace, and default parameters for Pajek.

To investigate the underlying molecular mechanisms, six gene expression datasets were retrieved from the Gene Expression Omnibus (GEO) database, comprising three periodontal disease datasets (GSE10334, GSE16134, GSE23586) and three placental disease datasets (GSE44711, GSE48424, GSE66273). Six GEO datasets were included based on the following criteria: (1) human tissue samples; (2) clear grouping of disease vs. control; (3) sample size ≥ 5 per group; (4) use of Affymetrix or Illumina platforms. Batch effects were adjusted for using the ComBat function in the sva package before differential expression analysis. Differentially expressed genes were identified applying thresholds of P < 0.05 and |log2FC| > 0.85. This relatively lenient log2FC threshold was selected to capture biologically relevant yet moderately altered genes, accounting for the subtle transcriptomic changes characteristic of chronic inflammatory oral and placental tissues, an approach supported by prior studies on similar tissues.

Subsequently, enrichment analyses were conducted on these common targets to uncover core regulatory pathways. We performed Gene Ontology (GO), Kyoto Encyclopedia of Genes and Genomes (KEGG), and Disease Ontology (DO) enrichment analyses with a significance threshold of FDR < 0.05 (Chang et al., 2025). These functional and pathway annotations collectively elucidated the key biological processes and molecular mechanisms underlying the crosstalk within the oral-placental axis.

### 2.3 Classification of disease types

This study focuses on the oral-placental axis hypothesis, aiming to clarify its disease spectrum, analyze standardized research trends, and establish a standardized classification system for related diseases in this field. The research integrates scientometric literature data, bioinformatic findings on molecular mechanisms, and clinical epidemiological classification criteria. First, keywords related to oral, placental, and diseases affecting pregnancy outcomes were extracted from eligible literature using the bibliometrix package in R, and a preliminary disease list was generated through standardized processing. Next, the dataset was augmented through cross-referencing results from Disease Ontology (DO) enrichment analysis and functional enrichment analysis of core intersecting genes, while the standardization of disease names and classification rationales was verified with reference to the Clinical Practice Guidelines for Periodontology and Obstetrics and Gynecology (9th Edition). Subsequently, guided by the core mechanisms linking oral pathological factors to placental function and perinatal outcomes, classification criteria were developed based on literature citation frequency, association strength, and consistency in clinical diagnosis. Diseases were divided into two main categories according to the primary site of lesion: oral-related diseases and placental/placenta-associated perinatal diseases, with subcategories further stratified by pathological characteristics and clinical manifestations. The final classification system was finalized by two independent researchers, with discrepancies resolved via consensus discussion or third-party arbitration, providing a standardized foundation for quantitative research, trend analysis, mechanistic investigations, and clinical diagnosis and treatment in this field.

### 2.4 Validation of core hub genes in the oral-placental axis

To validate the stability and clinical applicability of the core shared genes identified as associated with the oral-placental axis, two independent approaches were employed, namely quantitative real-time polymerase chain reaction (qRT-PCR) and validation of gene expression using external datasets from the Gene Expression Omnibus (GEO) database. For qRT-PCR validation, total RNA was extracted from clinical gingival and placental tissues using TRIzol reagent. Reverse transcription was conducted to synthesize cDNA, and qRT-PCR was performed using SYBR Green master mix on a real-time PCR system. GAPDH was used as an internal control, and relative expression levels were calculated using the 2−ΔΔCt method. For GEO dataset validation, gene expression profiles were obtained from two independent cohorts: GSE173078 (periodontal disease dataset) and GSE75010 (preeclampsia dataset). Differential expression analysis of core hub genes was conducted between disease and control groups. Expression distribution was visualized using boxplots, and the diagnostic efficiency was evaluated.

## 3 Results

### 3.1 General analysis

This bibliometric analysis included 196 eligible publications on the oral–placental axis from 2016 to 2025 (**Supplementary Figure 1**). These studies involved 8061 references, 1096 authors, and 136 journals. The field showed an annual growth rate of 5.03%, with a mean document age of 5.04 years, an average citation rate of 35.41 per article, and an international collaboration rate of 25%. The average number of co-authors per paper was 8.22, with only 5 single-author publications, indicating strong research collaboration.

**Figure 1:**
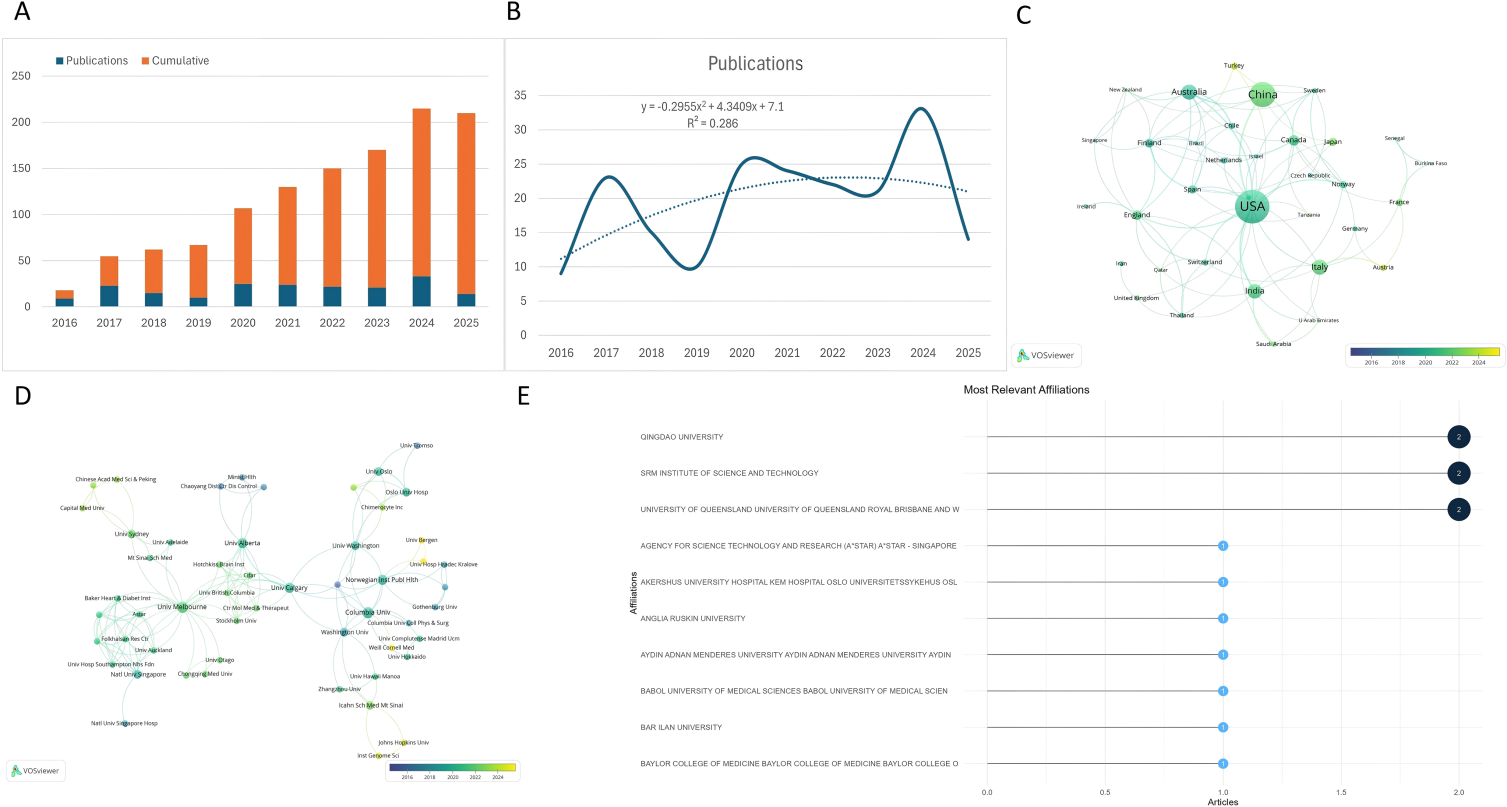
Research trends and collaboration patterns in the oral-placental axis: analysis of publication volume, country/institutional cooperation, and academic contributions. **(A)** Annual cumulative and yearly publication distribution, showing global changes in oral-placental axis-related literature output from 2016 to 2025; **(B)** Polynomial fitting curve of publication trends, reflecting the long-term development trajectory of this field (R²=0.286); **(C)** Temporal network diagram of country collaboration, with nodes representing countries and line thickness indicating collaboration strength; **(D)** Temporal network diagram of institutional collaboration, with nodes representing research institutions and line thickness reflecting cross-institutional collaboration frequency; **(E)** Ranking of the top 10 institutions by publication volume, with bar height corresponding to total publications per institution. OPA, Oral-placental axis.

Although annual output fluctuated, it exhibited an overall upward trend. Publications began with 9 in 2016, rose rapidly to 23 in 2017, then fluctuated between 10 and 15 from 2018 to 2019, before rebounding and stabilizing at 21–25 from 2020 to 2023. Output peaked at 33 articles in 2024 (16.84% of total), with 14 papers recorded in 2025 prior to the cutoff date. Cumulative publications increased continuously, and polynomial curve fitting (R² = 0.286) suggests that research on the oral–placental axis is gaining global attention and entering a phase of steady development (**Figures 1A, B**).

### 3.2 Distribution of country, institutions, authors, journals, references

In the international collaboration network analysis, the top 10 countries accounted for the majority of publication output in the field of oral-placental axis research, with the United States ranking first in the number of related publications (n=52) and China following closely (n=32) (**Supplementary Table 1**). **Figure 1C** and **Figure 1D** visualize the collaborative relationships among different countries and institutions via CiteSpace and VOSviewer. The United States, China, and Australia exhibited high publication impact in this field, which is likely attributable to their robust collaborative ties with other countries and research institutions across the globe.

**Supplementary Table 2** presents the top 10 institutions ranked by publication output, among which the University of Queensland stood out with the highest number of documents (n=7) and notable citation counts, and Brigham & Womens Hosp as well as Harvard Med Sch, despite having 2 publications each, achieved a total link strength of 18, reflecting their high academic influence. These findings suggest that the research quality of these core institutions is widely recognized by the academic community. **Figures 1D, E** depict the inter-institutional collaboration networks in this research field. These figures clearly show that the overall inter-institutional collaboration system is not yet fully established, implying that the depth and breadth of cooperation between different research institutions require further expansion and deepening. The University of Queensland emerged as the institution with the highest number of publications in the oral-placental axis research (n=7), followed by the University of Melbourne (n=4).

**Supplementary Table 3** lists the top 10 core authors ranked by the number of publications, among whom TUOMINEN, HEIDI from the University of Turku was the most productive author with 3 published articles and an H-index of 3. Meanwhile, GOMEZ-ARANGO, LUISA F. from the University of Queensland contributed 2 high-quality papers to the top 10 and obtained the highest citation count (182) among these authors in recent years, emerging as a representative scholar in the field (**Supplementary Tables 4** and **5**).

**Figure 2:**
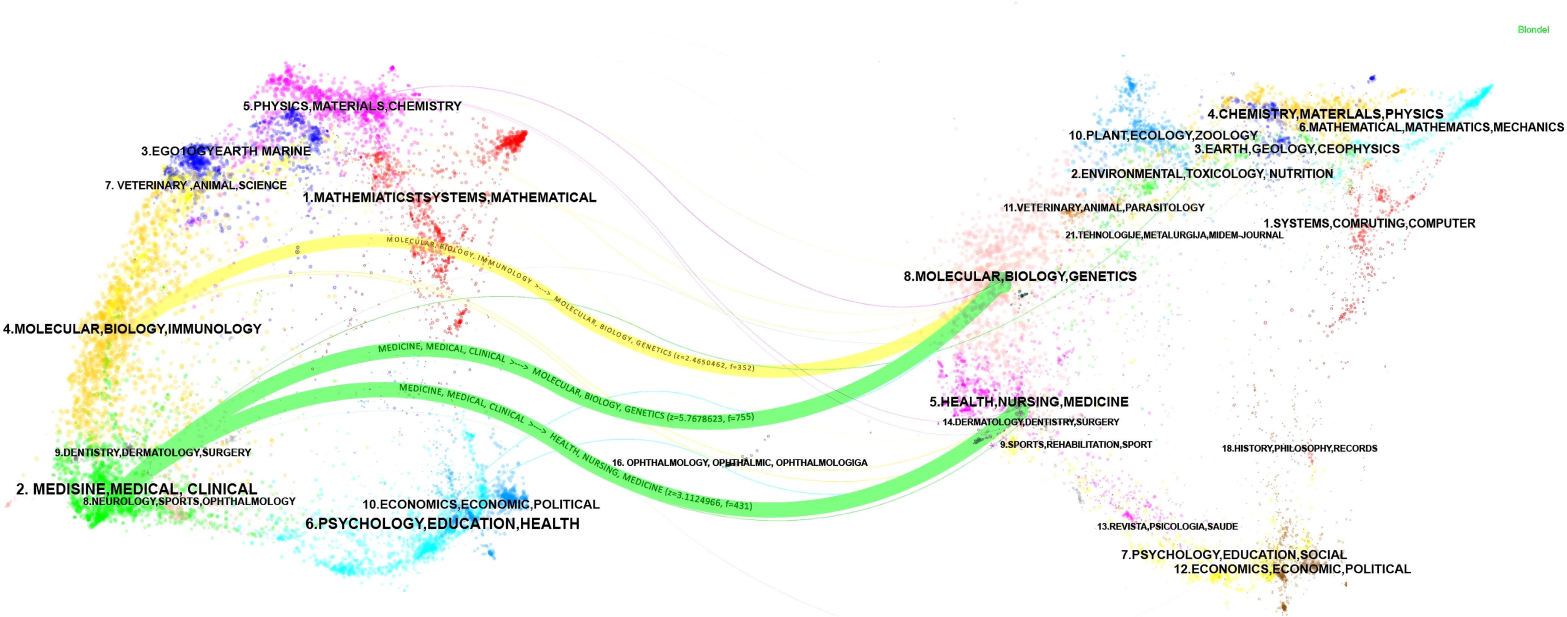
Bidirectional distribution map of oral-placental axis-related journals from 2016 to 2025. This map illustrates the interdisciplinary distribution of literature across five core disciplines: Obstetrics and Gynecology, Periodontology/Dentistry, Microbiology, Immunology, and Reproductive Medicine; node size represents total publications per journal, line thickness indicates inter-disciplinary citation frequency, visually presenting the intensity of cross-domain connections. OPA, Oral-placental axis.

**Supplementary Table 6** displays the top 10 most cited papers in the field of oral-placental axis research, which primarily focus on core scientific issues including the controversy regarding the existence of the placental microbiome, the mechanisms underlying the correlation between oral pathogenic bacteria and adverse pregnancy outcomes, and the vertical transmission pathway of oral-placental microbiota. **Figure 2** presents a bimap illustrating the journal coverage of literature on the oral-placental axis from 2016 to 2025. The bimap depicts the interdisciplinary distribution of publications across major subject categories: obstetrics/gynecology, periodontology/dentistry, microbiology, immunology, and reproductive medicine. Node size corresponds to the number of publications per journal, while link thickness represents cross-category citation frequency. **Supplementary Table 7** presents the detailed characteristics of the top 20 journals by publication volume in this field, including journal name, publisher, ISSN, impact factor, journal category, and citation metrics. These journals represent the core academic platforms for disseminating findings related to the oral-placental axis, as most are high-quality peer-reviewed publications in obstetrics, gynecology, dentistry, and microbiology, reflecting the multidisciplinary nature and academic influence of the research field.

### 3.3 Keyword analysis

The keyword co-occurrence analysis revealed the core terms and their interrelationships within the research field of the oral-placental axis. Keywords such as”Preterm birth”, “Periodontal diseases”and”Adverse pregnancy outcomes” frequently co-occurred in the literature, forming a dense network of closely related themes. In particular, the terms “Preterm birth”, “Periodontal diseases” and “Pregnancy” demonstrated strong linkages, indicating a sustained research focus on the interaction between oral pathological lesions and placental functional impairment in the context of pregnancy (**Supplementary Figure 2**). Additionally, several studies have expanded into mechanistic domains by incorporating terms such as “Inflammation” and “Oral microbiome”, suggesting a deepening of research from clinical phenotype correlation toward microbial and molecular pathogenesis underlying the oral-placental axis crosstalk.

**Figure 3:**
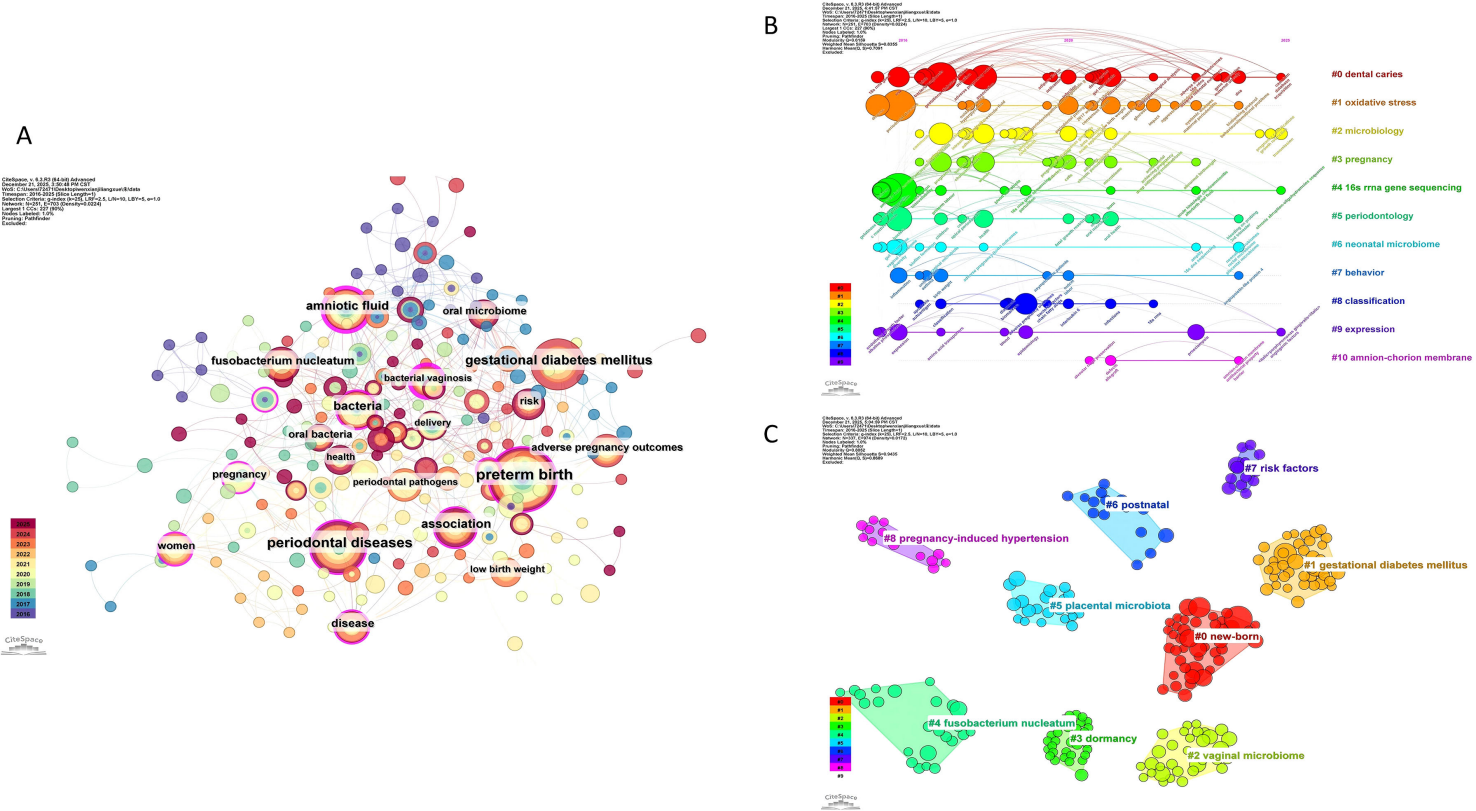
Keyword co-citation and cluster analysis in oral-placental axis research. **(A)** Keyword co-occurrence network, with nodes representing high-frequency keywords, lines indicating co-occurrence relationships, and colors distinguishing cluster themes, reflecting research hotspot association patterns; **(B)** Linear temporal evolution analysis of keywords, showing the temporal fluctuations of core keywords from 2016 to 2025, revealing dynamic shifts in research focus; **(C)** Core clusters and representative literature in the co-citation network, annotating cluster themes, core keywords, and highly cited literature, clearly presenting the knowledge structure of the field. OPA, Oral-placental axis.

A longitudinal analysis of keyword frequency trends further illustrated the dynamic evolution of research themes over time. The findings indicate that the preterm birth cluster and the periodontal diseases cluster consistently retained dominance regarding both node magnitude and temporal persistence (**Figure 3A**). In contrast, the gestational diabetes mellitus cluster has shown a rising trend in recent years, reflecting a growing interest in mechanism-based research targeting specific pregnant populations and the oral-placental metabolic axis. Meanwhile, early-stage keywords such as “Diversity”(microbial diversity) have steadily declined in relevance, signaling a gradual shift in focus from basic microbial community characterization to the in-depth exploration of specific oral pathogenic bacteria and their placental invasion mechanisms. Notably, “Amniotic fluid” established multiple strong connections with various core research clusters due to its highest centrality in the keyword network, underscoring its pivotal bridging role in the oral-placental axis interaction framework (**Figure 3B**).

**Figure 4:**
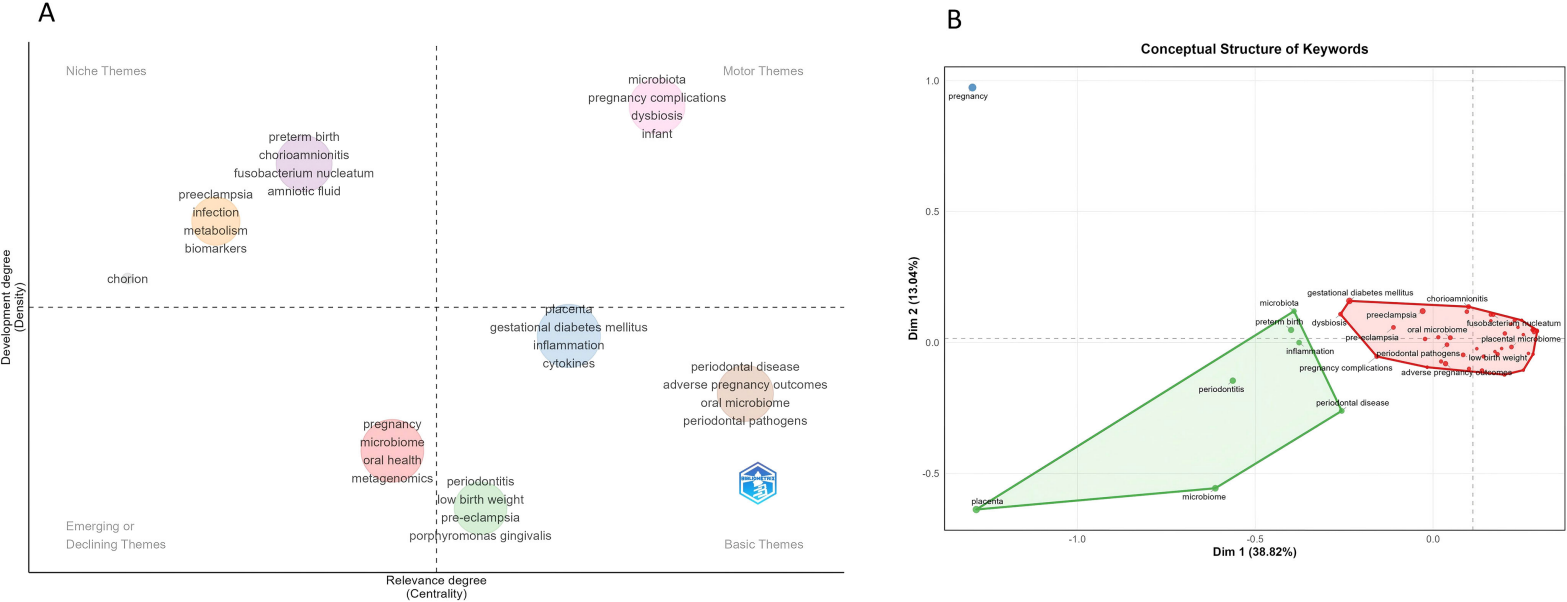
Analysis of research themes and conceptual structure in the oral-placental axis. **(A)** Strategic distribution map of research themes: The horizontal axis represents centrality (field importance), and the vertical axis represents density (internal maturity); the first quadrant indicates mature core themes, the second quadrant peripheral secondary themes, the third quadrant emerging/declining themes, and the fourth quadrant underdeveloped themes, facilitating the assessment of theme development stages; **(B)** Keyword conceptual structure map, with the outer layer containing clinical outcome-related keywords, the middle layer keywords related to microbial/inflammatory mechanisms, and the core keywords related to pregnancy fundamentals, clearly presenting the hierarchical conceptual relationships in the field. OPA, Oral-placental axis.

The clustering analysis of keyword co-occurrence provided further insight into the structural composition of research topics (**Figure 3C**). Several distinct thematic clusters were identified, with the “Preterm birth-Periodontal diseases” cluster emerging as the largest, followed by the “Oral microbiome-Placental microbiome” cluster and the “Inflammation-Pathogenic bacteria” cluster. These clusters highlight the central role of oral disease-induced adverse pregnancy outcomes and their close association with microbial translocation and inflammatory response in oral-placental axis research. A strategic thematic map categorized research topics into four quadrants, illustrating the shifting focus from initial clinical phenotype correlation toward mechanistic exploration and clinical translational research (**Figure 4A**). Contemporary hotspots included themes such as “Oral microbiome”, “Pre-eclampsia” and “Low birth weight”, while earlier themes such as “Vaginal microbiome” and “Delivery” gradually declined in prominence, reflecting the field’s evolution toward more precise and in-depth mechanistic and population-specific research. The conceptual structure map derived from core keywords further elucidated the semantic organization of research in this domain, providing a visual taxonomy of how core concepts interrelate—with clinical outcome-oriented keywords as the outer layer, microbial and inflammatory mechanistic keywords as the middle layer, and basic pregnancy background keywords as the core (**Figure 4B**).

**Figure 5:**
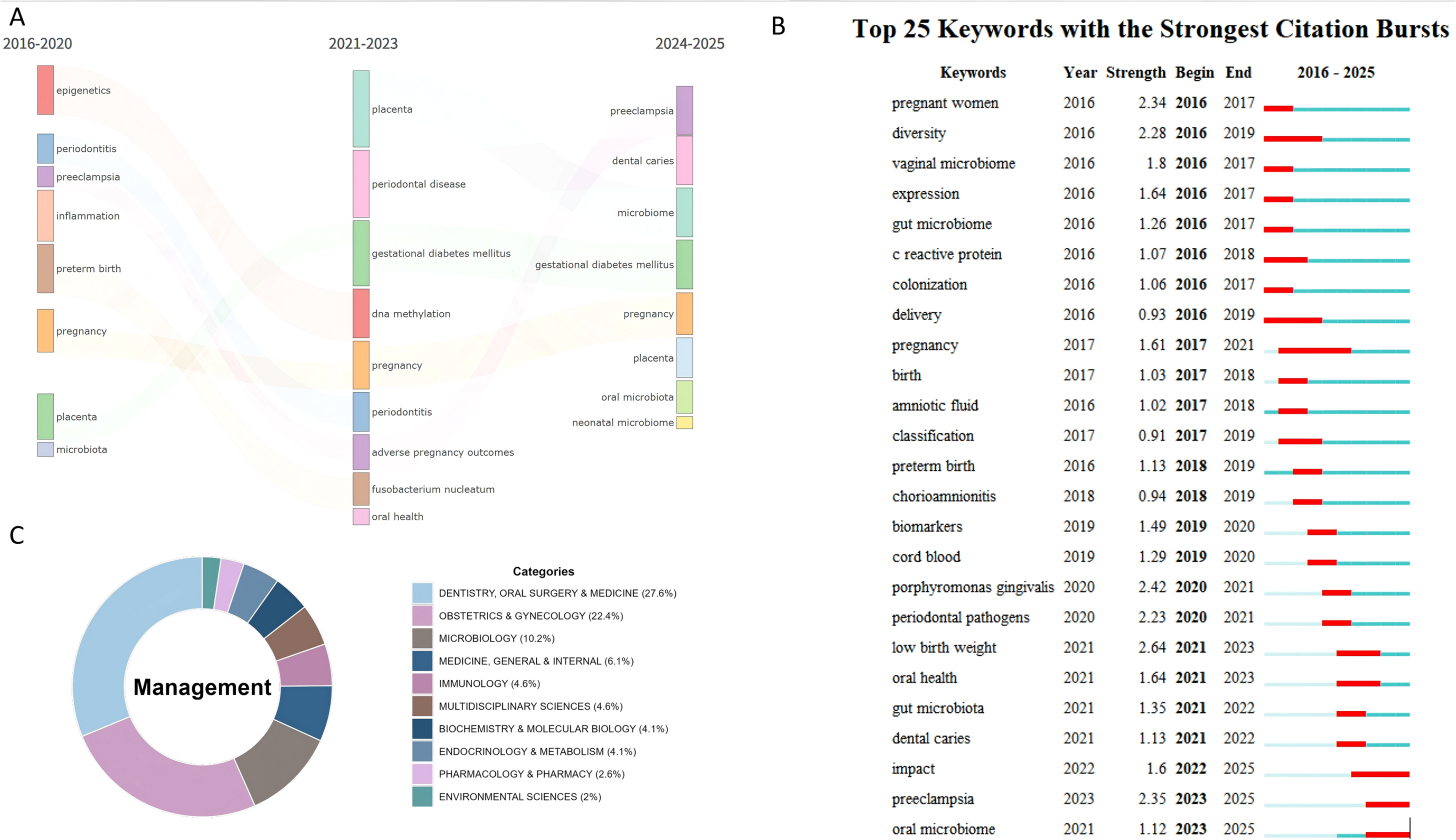
Keyword covariance, burst intensity, and thematic distribution of highly cited literature in the oral-placental axis. **(A)** Keyword covariance network diagram, with node association strength reflecting keyword co-occurrence stability, and colors distinguishing four major research clusters: Clinical phenotype association, Microbial pathogenesis, Pathological mechanisms, and Translational research; **(B)** Temporal chart of the top 30 burst keywords, with bar height indicating burst intensity and the horizontal axis representing time span, annotating hotspots at different stages (e.g., low birth weight, preeclampsia), reflecting the phased evolution of research hotspots; **(C)** Pie chart of thematic proportions among the top 100 most cited articles, showing the distribution of four major themes: Placental microbiota, Oral pathogens, Inflammatory mechanisms, and Clinical interventions. OPA, Oral-placental axis.

A comprehensive analysis of the keyword co-occurrence network identified four main thematic clusters, each represented by a distinct color (**Figure 5A**). The first cluster focused on the intricate interconnections between oral diseases and placental dysfunction, exploring clinical phenotype correlations centered on periodontal disease and various adverse pregnancy outcomes such as preterm birth and low birth weight. The second cluster was microbiota-oriented, addressing specific oral pathogenic bacteria such as Porphyromonas gingivalis and Fusobacterium nucleatum, their placental detection characteristics and vertical transmission pathways via hematogenous dissemination. The third cluster concentrated on pathophysiological mechanisms of the oral-placental axis, such as inflammatory factor-mediated signal transduction, maternal-fetal immune microenvironment crosstalk and placental tissue damage repair. Finally, the fourth cluster encompassed translational research themes, including oral health intervention during pregnancy, risk assessment of adverse pregnancy outcomes and targeted inhibition of oral pathogenic bacterial invasion. Together, these clusters reflect the structural evolution of the oral-placental axis research landscape and provide direction for future multidisciplinary investigations integrating oral medicine, obstetrics and gynecology, microbiology and immunology.

The keyword burst analysis identified the most influential terms in the field based on surge intensity and duration (**Figure 5B**). Among the top burst keywords, “Low birth weight” exhibited the strongest burst strength with a score of 2.64 during its active period, marking it as the most concentrated research focus in that phase, while “Pregnancy” maintained the longest burst duration, serving as a persistent basic research core. Research themes have undergone distinct transitions: from an early emphasis on clinical phenotype correlation and basic microbial community analysis (2016–2019), to specific pathogenic bacteria and inflammatory mechanism research (2019–2022), and more recently to precision population research and emerging adverse pregnancy outcome exploration centered on pre-eclampsia (2023–2025). Among the top cited articles in the field, a vast majority focus on the placental microbiome and its correlation with oral microbiota (**Supplementary Figure 2**), and nearly 40% of the high-citation literature centers on the impact of oral pathogenic bacteria on placental function and adverse pregnancy outcomes, highlighting the core research focus and academic recognition of microbial crosstalk in the oral-placental axis (**Figure 5C**).

### 3.4 Genetic cross-analysis of oral-placental axis

**Figure 6:**
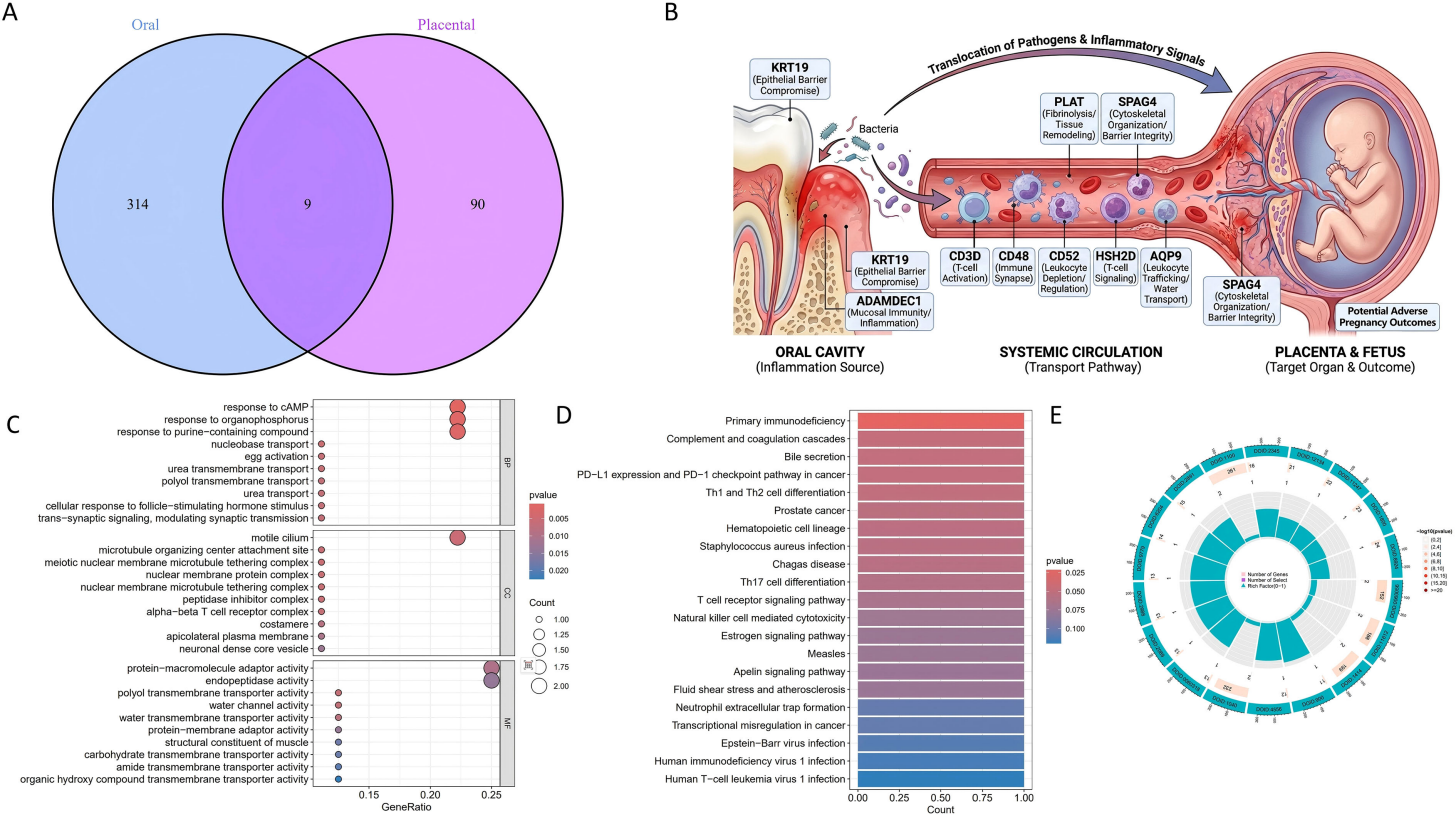
Cross-overlapping gene analysis in the oral-placental axis. **(A)** Venn diagram of differential gene overlap between oral diseases and placental pathologies, showing the number of shared differential genes between periodontal disease and placental disorders; **(B)** Scatter plot depicting the screening of overlapping genes based on thresholds, with criteria of P<0.05 and |log2FC|>0.85, highlighting core hub genes (KRT19, AQP9, PLAT, SPAG4); **(C)** Bubble plot of KEGG pathway enrichment, with bubble size representing gene count and color indicating enrichment significance (FDR<0.05), core enriched pathways including Primary immunodeficiency and Complement and coagulation cascades; **(D)** Bar chart of GO functional enrichment, showing the enrichment distribution of overlapping genes in biological processes such as cell proliferation and migration, epithelial barrier function, and immune activation; **(E)** Disease enrichment network diagram (DO), with nodes representing diseases and lines indicating gene associations, demonstrating the associations between overlapping genes and conditions such as oral mucosal diseases, placental dysfunction, and pregnancy vascular diseases. DO, Disease Ontology; KEGG, Kyoto Encyclopedia of Genes and Genomes; GO, Gene Ontology; OPA, Oral-placental axis.

Gene crossover studies identified a set of shared co-expressed genes between oral diseases and placental lesions, with SPAG4, AQP9, KRT19 and PLAT as the core shared genes (**Figure 6A**). The results revealed that genes such as AQP9, KRT19, PLAT and CD52 play critical roles across both oral disease and placental lesion-related molecular pathways, suggesting shared regulatory mechanisms in tissue damage, immune response and metabolic regulation between oral and placental tissues (**Figure 6B**, **Supplementary Tables 8**, **9**).

KEGG analysis showed that the identified shared genes were significantly enriched in pathways related to immune regulation, tissue remodeling, vascular function and inflammatory response, which are the core pathophysiological pathways mediating crosstalk within the oral-placental axis (**Figure 6C**). GO analysis indicated that the identified shared genes play pivotal roles in cell proliferation and migration, epithelial barrier function, metabolic reprogramming, immune cell activation and extracellular matrix remodeling, which are closely associated with the structural and functional homeostasis of oral and placental tissues (**Figure 6D**). DO analysis further revealed significant associations between the shared genes and oral mucosal diseases, placental dysfunction-related disorders, pregnancy vascular diseases and inflammatory tissue lesions, confirming the direct molecular link between oral pathological changes and abnormal placental function (**Figure 6E**).

### 3.5 Genetic and microbial mechanisms of the oral-placental axis

In addition to genetic crossover analyses, we further elucidated the core molecular and microbial mechanisms underlying the oral-placental axis interaction, with oral microbiota and their derived factors identified as key mediators of placental dysfunction and adverse pregnancy outcomes. Sequence homology analysis confirmed high genetic similarity between oral pathogenic bacteria (e.g., Fusobacterium nucleatum, Porphyromonas gingivalis) and bacteria isolated from placental tissues (**Supplementary Figure 3A**), verifying the direct translocation of oral microbes to the placenta. **Supplementary Table 10** provides a statistical summary of co-occurring bacterial species identified in both oral and placental microbiota, listing dominant genera, species, detection rate in oral samples, detection rate in placental samples, and association strength with adverse pregnancy outcomes. This table systematically demonstrates the overlapping microbial composition between the oral cavity and placenta, providing direct microbiological evidence for the microbial transmission pathway of the oral-placental axis. Hematogenous dissemination of oral microbial components and metabolites triggers the activation of the NF−κB signaling pathway and downstream inflammatory cascades in placental tissue, leading to placental inflammation, trophoblast dysfunction and impaired maternal-fetal immune tolerance (**Supplementary Figure 3B**). Furthermore, a meta-analysis with 95% confidence intervals further quantified the significant association between oral pathogenic bacteria and major adverse pregnancy outcomes, including preterm birth, low birth weight and gestational diabetes mellitus (**Supplementary Figure 3C**). Temporal and disciplinary evolution analysis revealed a steady growth in annual publication output from 2016 to 2025, with burst detection identifying preterm birth, periodontal diseases and gestational diabetes mellitus as the earliest and most sustained research hotspots, followed by a shift to mechanistic exploration focused on oral microbiome, placental epigenetics and inflammatory mediators (**Supplementary Figure 4A**). Furthermore, disciplinary knowledge flow mapping demonstrated that the field is an interdisciplinary integration of stomatology, obstetrics and gynecology, microbiology and immunology (**Supplementary Figure 4B**). Cross-country collaboration networks featured a core-peripheral structure, with the USA, China and Australia as global core nodes (**Supplementary Table 11**), while core research institutions formed three main research paradigms (translational, population clinical, basic mechanistic research) (**Supplementary Table 12**). Under both physiological and pathological conditions, the oral cavity and placenta form a bidirectional regulatory axis through microbial translocation, systemic inflammation, immune crosstalk and genetic regulation (**Supplementary Figure 5**), where pathological states such as periodontitis disrupt this axis to cause adverse pregnancy outcomes and vice versa. Shared pathological processes and core molecular linkages form the basis of this interaction. This study provides a comprehensive perspective on the multi-dimensional mechanisms of the oral-placental axis, offering a theoretical basis for coordinated prevention and management strategies.

### 3.6 Validation of core hub genes

**Figure 7:**
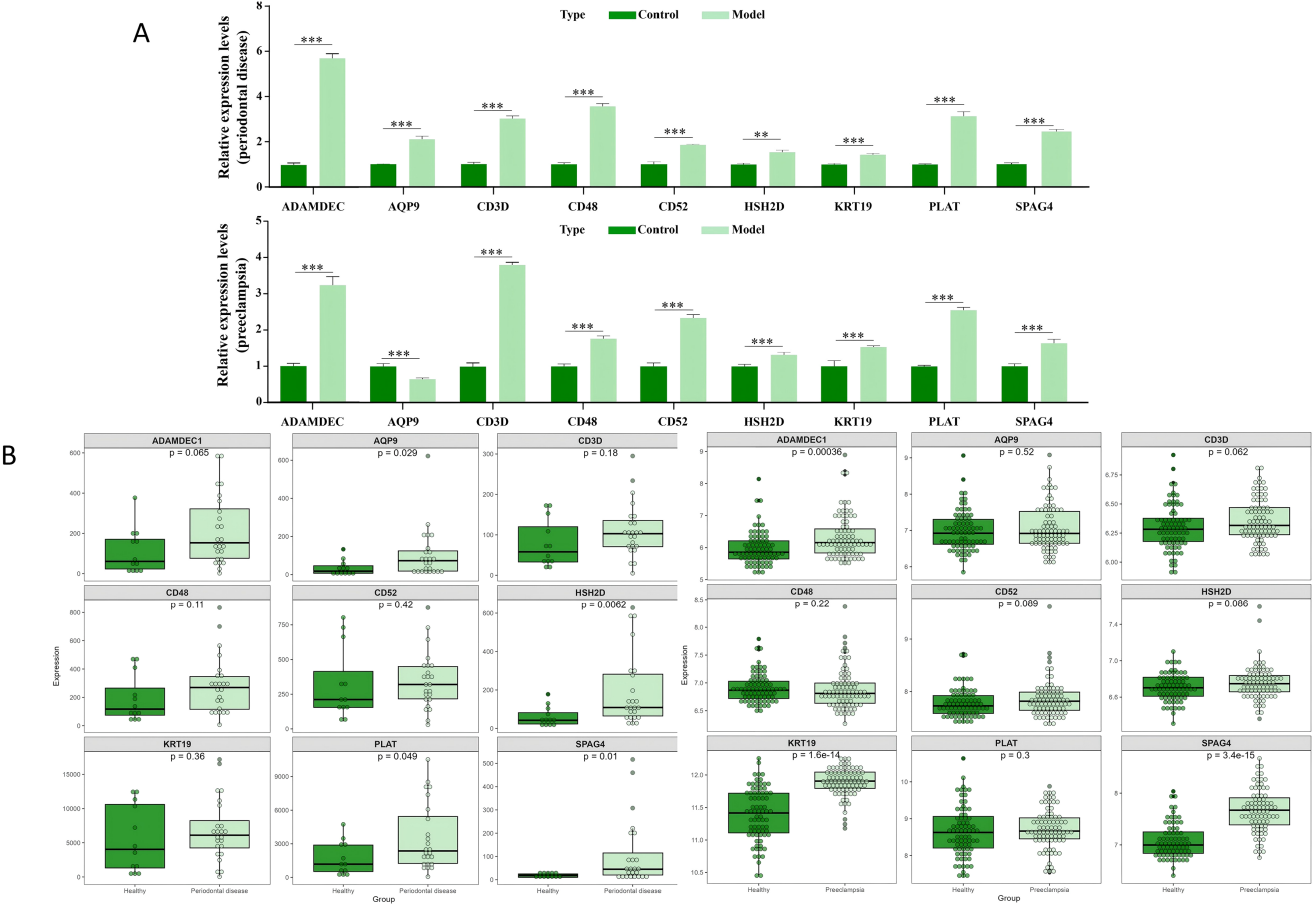
Validation of core hub genes in the oral-placental axis. **(A)** Clinical sample qRT-PCR validation: Bar charts showing relative expression levels of core genes (KRT19, ADAMDEC1, AQP9, SPAG4, PLAT) in periodontal disease tissues and preeclampsia placental tissues, which were all significantly upregulated (*P<0.05, **P<0.01, ***P<0.001), with GAPDH as the reference gene; **(B)** Independent validation using GEO datasets: Box plots respectively showing the expression distribution of core genes in the periodontal disease dataset GSE173078 and the preeclampsia dataset GSE75010, with significantly higher expression in the disease group compared to the control group, consistent with bioinformatics predictions.

To validate the robustness of core shared genes in the oral-placental axis, we conducted qRT-PCR on clinical specimens and performed external validation using GEO datasets. **Figure 7A** demonstrates that the core hub genes (KRT19, ADAMDEC1, AQP9, SPAG4, PLAT) were significantly upregulated in periodontal lesions and preeclamptic placental tissues relative to the respective control groups (all P < 0.05). Validation using GEO datasets (**Figure 7B**) revealed consistent expression trends. In the GSE173078 dataset (periodontal disease), the core hub genes exhibited significant upregulation in affected tissues. In GSE75010 (preeclampsia), these genes were also markedly increased in preeclamptic placenta. The expression patterns were highly consistent with our bioinformatic predictions.

## 4 Discussion

Oral health constitutes an indispensable and underappreciated cornerstone of maternal–fetal health, and the oral–placental axis (OPA) hypothesis has matured into a landmark interdisciplinary paradigm at the interface of stomatology, obstetrics and gynecology, microbiology, and immunology. Adverse pregnancy outcomes (APOs), including preterm birth (PTB), low birth weight (LBW), preeclampsia (PE), and gestational diabetes mellitus (GDM), continue to dominate the global burden of perinatal morbidity and mortality, imposing severe short−term clinical risks and long−term physical, psychological, and socioeconomic burdens on mothers, infants, and healthcare systems worldwide (Banerjee et al., 2021; Ross et al., 2023). Over recent decades, converging epidemiological, clinical, and experimental evidence has consistently linked chronic oral inflammatory diseases—especially periodontal diseases—to placental dysfunction and adverse perinatal events, thereby solidifying the theoretical foundation of the OPA hypothesis (Beckers and Sones, 2020; Boggess, 2020). Nevertheless, the causal relationship between oral disorders and APOs, the detailed molecular and microbial mechanisms governing inter−tissue crosstalk, and the translational inconsistency of periodontal intervention trials remain incompletely elucidated and highly debated. These critical gaps substantially restrict the translation of scientific insights into standardized, evidence−based perinatal oral care strategies (Guan et al., 2023). Against this background, the present study integrated scientometric visualization and bioinformatics analysis to systematically dissect the global research status, evolutionary trends, knowledge structure, and core molecular–microbial mechanisms of the OPA from 2016 to 2025, aiming to clarify consensus, resolve controversies, and propose forward−looking directions for the field.

Global research output related to the OPA displayed a stable annual growth rate of 5.03%, accompanied by a marked publication peak in 2024, indicating that this field has entered a phase of steady, high−quality development with continuously expanding global academic attention. This upward trend is closely associated with the worldwide emphasis on preconception and perinatal health, as well as rapid technological advances in high−throughput sequencing, multi−omics integration, and bioinformatic mining, which have collectively enabled deeper mechanistic exploration. The international collaboration rate reached 25%, indicating that cross−national and cross−institutional cooperation has become an important driver of progress; however, the overall depth and breadth of collaboration remain insufficient, with considerable potential for improvement (Sarkar et al., 2024). The United States, China, and Australia emerged as the top three contributing countries, a pattern attributed to their robust research funding, well−established clinical cohort platforms, and extensive academic exchange networks (Rowe and Rosch, 2021). Among research institutions, the University of Queensland ranked first in publication volume and demonstrated outstanding academic influence, having made pioneering contributions to clarifying microbial vertical transmission pathways and evaluating clinical intervention strategies (Kitamoto et al., 2020). These results collectively reveal a globally interconnected yet regionally concentrated research landscape, with a small number of core countries and institutions leading paradigm shifts and methodological innovations.

Keyword co−occurrence, cluster analysis, and burst detection collectively delineated a clear, stage−wise evolutionary trajectory of research hotspots in the OPA field. From 2016 to 2019, studies were dominated by clinical phenotype association analyses, focusing on the statistical association between periodontal diseases and adverse pregnancy outcomes, especially PTB and LBW (Devraj et al., 2025). Between 2019 and 2022, the research focus shifted to mechanistic dissection, with growing attention to the oral microbiome, specific pathogenic bacteria, and inflammatory signaling networks. Since 2023, emerging hotspots have centered on GDM−related metabolic crosstalk, PE, and precision translational research, signifying a strategic shift from descriptive correlation to mechanism−driven clinical application (Ajaz et al., 2025). Notably, the relevance of broad terms such as microbial diversity gradually declined, while investigations into *Fusobacterium nucleatum* and *Porphyromonas gingivalis* gained prominence, reflecting a field−wide transition from broad community profiling to pathogen−specific mechanistic validation (Gan et al., 2025). Furthermore, the keyword “amniotic fluid” exhibited high centrality in the co−occurrence network, suggesting its pivotal role as a biological conduit for microbial translocation and inflammatory signal transmission in the OPA (Sacco et al., 2008). Four well−defined thematic clusters were identified: clinical phenotype correlation, microbial ecology and pathogenicity, pathophysiological mechanisms, and translational research. Together, these clusters form a complete, hierarchical research system extending from clinical observation to mechanistic exploration and translational application, providing a structured framework to guide future investigations (Holtmann et al., 2025).

Bioinformatic integrative analysis systematically elucidated the conserved molecular basis underlying the crosstalk between oral inflammatory lesions and placental dysfunction. A set of core shared genes was identified, including *KRT19*, *ADAMDEC1*, *AQP9*, *SPAG4*, and *PLAT*, which exert critical regulatory functions in tissue damage repair, immune response, epithelial barrier integrity, and metabolic homeostasis across both oral and placental tissues (Du et al., 2024; Jordan et al., 2024). KEGG enrichment analysis revealed that these overlapping genes were significantly enriched in immune−related pathways, including primary immunodeficiency and complement and coagulation cascades, which are recognized as key pathophysiological mechanisms mediating OPA−driven adverse outcomes (Ermakov et al., 2025). This finding strongly supports the indirect pathway of the OPA hypothesis, in which systemic inflammation induced by oral infection acts as a critical remote mediator of placental impairment (Escobar-Arregoces et al., 2018). GO enrichment analysis further confirmed the involvement of shared genes in cell proliferation and migration, extracellular matrix remodeling, immune cell activation, and metabolic reprogramming—biological processes essential for maintaining structural and functional homeostasis in oral and placental tissues (Tolg et al., 2020). Disease Ontology (DO) analysis identified significant molecular associations between oral mucosal diseases, placental dysfunction−related disorders, pregnancy vascular diseases, and inflammatory lesions, providing robust molecular evidence for the causal association between oral pathological changes and placental injury (Kim et al., 2020). Further pathway screening revealed that the Apelin signaling pathway and T cell receptor signaling pathway serve as additional key regulatory nodes, integrating inflammatory, oxidative stress, and signals related to placental vascular function into a complex molecular network (Nakashima et al., 2024).

Analyses of microbial mechanisms provided conclusive evidence supporting the direct translocation pathway of the OPA. Sequence homology verification confirmed high genetic similarity between oral pathogenic bacteria (including *Fusobacterium nucleatum* and *Porphyromonas gingivalis*) and strains isolated from placental tissues, directly validating the hematogenous spread of oral microbes to the placental microenvironment (Schenkein et al., 2020; Wang et al., 2024). The consistent detection of these pathogens in cord blood and placental tissues of preterm infants offers robust *in vivo* clinical evidence for this direct route (Seliem and Sultan, 2018). In parallel, microbial components, metabolites, outer membrane vesicles, and infected macrophage−derived extracellular vesicles that enter the systemic circulation can activate the NF−κB signaling cascade in placental tissue, triggering local inflammation, trophoblast dysfunction, and impaired maternal–fetal immune tolerance (Pockpa et al., 2021). Importantly, the direct and indirect pathways do not function independently but interact synergistically: pathogen−induced placental inflammation amplifies systemic immune activation, while systemic inflammation compromises placental barrier integrity, increasing susceptibility to further microbial invasion. This feed−forward vicious cycle exacerbates placental dysfunction and significantly elevates the risk of APOs, forming a unified, multi−modal mechanistic model of the OPA (Lin et al., 2022).

Notably, the proposed bidirectional regulatory model of the oral-placental axis serves primarily as a theoretical framework and roadmap for future research. In this study, only core shared molecular signatures (e.g., KRT19, AQP9, immune/inflammatory pathways) were empirically supported by bioinformatic analyses and experimental expression validation. Components involving reciprocal hormonal and microbial feedback loops were derived from a synthesis of the existing literature and require further experimental validation using *in vitro* and *in vivo* models.

The robustness of the present study was strengthened by dual validation including clinical qRT-PCR and independent GEO dataset expression confirmation. The core hub genes *KRT19, ADAMDEC1, AQP9, SPAG4*, and *PLAT* exhibited consistently upregulated expression in periodontal disease and preeclampsia in both clinical samples and public datasets (GSE75010 and GSE173078), thereby substantiating their key roles in the oral-placental axis crosstalk. *KRT19* plays a critical role in maintaining epithelial structural integrity, and its abnormal expression suggests impaired barrier function in both oral mucosa and placenta. *AQP9* participates in water transport and inflammatory response, which bridges local oral infection and systemic placental injury. *ADAMDEC1* mediates extracellular matrix degradation and immune activation, aggravating tissue damage in periodontium and placenta. *SPAG4* and *PLAT* are involved in cell migration and vascular homeostasis, with their dysregulation contributing to placental dysfunction and adverse pregnancy outcomes. The strong concordance among bioinformatic analysis, clinical validation, and external dataset verification bolsters the reliability of our findings. These core genes may serve as potential diagnostic biomarkers and intervention targets for oral health-related adverse pregnancy outcomes.

Persistent inconsistencies in the results of periodontal intervention randomized controlled trials (RCTs) represent a major bottleneck hindering clinical translation. Most high−quality RCTs indicate that non−surgical periodontal treatment performed in the second trimester fails to significantly reduce the incidence of APOs (Castro Dos Santos et al., 2024; Martins et al., 2025). This phenomenon can be explained by two key factors: first, the intervention window is excessively late, as oral pathogens have already colonized the placenta and caused irreversible functional damage before the second trimester, rendering subsequent treatment unable to reverse established placental injury (Caneiro-Queija et al., 2019b); second, marked heterogeneity exists in study design, including population selection criteria, treatment intensity and duration, and pregnancy outcome definitions, leading to poor reproducibility and incomparability across trials (Vera-Ponce et al., 2025). These findings strongly support a paradigm shift toward preconception oral health intervention as the optimal strategy. Eliminating oral infectious burdens before pregnancy can prevent pathogen translocation and placental damage at the earliest developmental stage, thereby maximizing the protective effect on perinatal outcomes. Additionally, the development of unified, standardized clinical trial protocols and outcome evaluation systems is urgently required to resolve translational controversies and facilitate evidence synthesis.

Advancements in microbiome and metabolic research have expanded the scope of the OPA to include two emerging sub−axes: the oral microbiome–placental microbiome axis and the oral–placental metabolic axis (Huang et al., 2023). Pregnancy−related hormonal and immunological changes disrupt oral microbial homeostasis, enabling pathobionts to spread hematogenously and reshape the composition and function of the placental microbiome, thereby modulating placental development and pregnancy outcomes (Le et al., 2021). Parallel investigations centered on GDM have revealed extensive metabolic crosstalk linking oral dysbiosis, insulin resistance, and placental metabolic dysfunction. These emerging axes enrich the multi−dimensional theoretical architecture of the OPA, transforming it from a narrow infection−mediated pathway into a comprehensive systems−biology framework that integrates microbial, immune, and metabolic signals.

Notably, the existence and functional relevance of the placental microbiome remain highly controversial. While several studies have identified microbial signatures in placental tissues, others contend that these findings likely reflect contamination rather than true commensal communities. This controversy underscores the need for rigorous negative controls and standardized sampling protocols in future studies elucidating microbial transmission along the oral-placental axis.

This study has several limitations that should be acknowledged. First, the scientometric analysis was restricted to English original articles and reviews, potentially introducing language and geographic bias and excluding valuable non−English literature. Second, bioinformatic predictions of core shared genes and signaling pathways require experimental validation using *in vitro* cell models and *in vivo* animal studies to confirm functional relevance and regulatory details. Third, the standardized disease classification system requires prospective validation in large−sample clinical cohorts to assess clinical applicability and feasibility. Fourth, the 10−year observation window limits the assessment of long−term evolutionary trends. Despite these limitations, the integrated scientometric–bioinformatic design upholds high objectivity, comprehensiveness, and scientific rigor, yielding reliable insights into the current state and future directions of the field.

In the context of global prioritization of perinatal health, future research in the OPA field should focus on six strategic priorities: (1) Initiate large−sample, multi−center, prospective cohort studies to robustly establish causality and develop standardized clinical evaluation indicators and diagnostic criteria; (2) Conduct in−depth mechanistic experiments to validate the functions of core hub genes and key signaling pathways and identify actionable molecular intervention targets; (3) Conduct preconception oral health intervention RCTs to evaluate the efficacy of early oral care in reducing APO risk; (4) Strengthen research on the oral microbiome–placental axis and oral–placental metabolic axis to refine mechanistic taxonomy and expand theoretical boundaries; (5) Promote deep international and cross−institutional collaboration to share clinical data, research resources, and technological platforms; (6) Integrate artificial intelligence and multi−omics technologies to construct APO predictive models based on oral health indicators, enabling personalized precision perinatal oral health management.

## 5 Conclusion

In summary, global research on the oral–placental axis has entered a stage of steady, high−quality development over the past decade, with a clear evolutionary trajectory from clinical phenotype correlation to in−depth mechanistic dissection and clinical translational research. The United States, China, and Australia serve as the primary drivers of global progress, while the University of Queensland and other leading institutions have made landmark contributions to establishing paradigms and clarifying mechanisms. At the molecular level, the core shared genes including *KRT19, ADAMDEC1, AQP9, SPAG4*, and *PLAT*, together with key immune− and inflammatory pathways such as primary immunodeficiency and complement and coagulation cascades, form the conserved molecular foundation of oral–placental crosstalk. At the microbial level, key pathogens including *Fusobacterium nucleatum* and *Porphyromonas gingivalis* mediate adverse pregnancy outcomes through two synergistic routes: direct hematogenous translocation and indirect systemic inflammatory activation. These pathways are further complemented by extracellular vesicle−mediated signal transmission, forming a complex, multimodal regulatory network. Notably, this study confirms that the oral–placental axis operates as a bidirectional regulatory system governed by microbial transmission, immune crosstalk, metabolic interplay, and shared genetic programs, substantially expanding and refining the classical hypothesis.

Inconsistent results from mid−gestation periodontal intervention RCTs highlight the urgent need for preconception oral health intervention and rigorous, standardized clinical trial design. Future research should prioritize clarifying causal relationships, validating molecular intervention targets, evaluating early preventive strategies, deepening microbiome and metabolic axis investigations, strengthening global collaborative networks, and leveraging innovative technologies to advance precision perinatal care. Collectively, these efforts will facilitate the formal integration of oral health assessment and early intervention into routine preconception and perinatal management, thereby providing a robust theoretical foundation and evidence−based clinical strategy for reducing the global burden of adverse pregnancy outcomes and improving maternal–fetal health.
