# Supplementary material for: A scientometric analysis of research related to the ‘oral-placental axis’ hypothesis: current status, hotspots, and future directions
Source: Front Cell Infect Microbiol. 2026 Jun 30;16:1864022. doi: 10.3389/fcimb.2026.1864022 (PMC13364982; doi:10.3389/fcimb.2026.1864022)
Supplement: Supplementary file 1 [file Presentation1.pdf]

frontiers

Supplementary Material

Supplementary Figures

Figure S1： Flowchart of Literature Screening for Oral-Placental Related Studies from 2016 to 2025. This figure illustrates the complete process of literature retrieval, deduplication, and screening: initial searches in Web of Science, Scopus, and PubMed yielded 808, 1,080, and 162 records, respectively; after deduplication, preliminary screening (excluding animal experiments, non-English articles, and non-original/review articles) and secondary screening, 196 eligible articles were finally included; the number of articles at each stage and reasons for exclusion are specified to ensure the representativeness and scientific validity of the study sample.

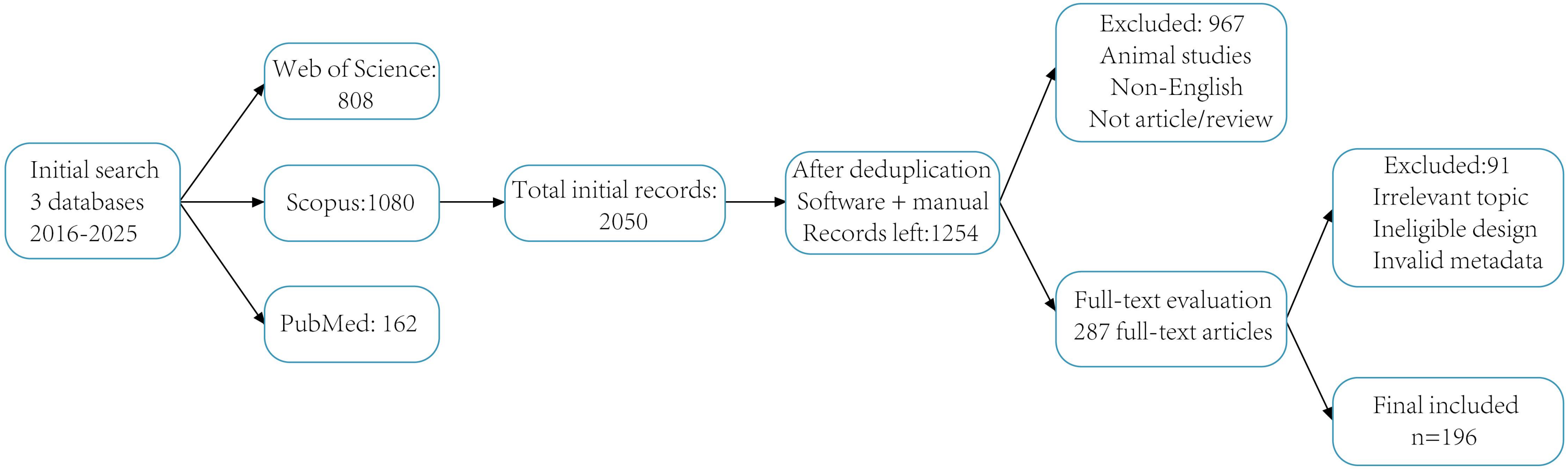

Figure S2： Subnetwork Map of Topic Distribution among the Top 100 Highly Cited Articles in Oral-Placental Research . (A) Subgraph of topic distribution for the top 100 highly cited papers on the oral-placental axis; (B) Annual publication output and keyword burst analysis of oral-placental axis research.

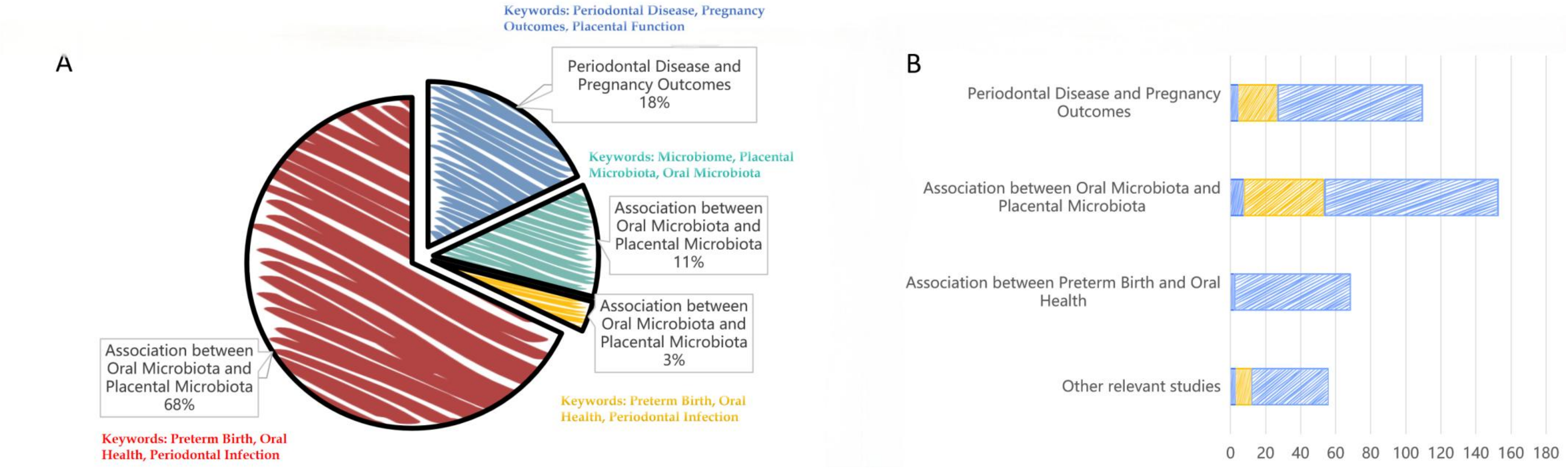

Figure S3： Molecular Mechanisms of the Association between Oral Microbiota and Placental Microbiota. (A) Gene sequence homology analysis of oral pathogens (Fusobacterium nucleatum, Porphyromonas gingivalis, etc.) and placental isolates confirms high genetic similarity, providing direct evidence for hematogenous transmission of oral microorganisms; (B) Oral microbial components/metabolites migrate to the placenta via the bloodstream, activating NF-κB and downstream inflammatory pathways, leading to placental inflammation, trophoblast dysfunction, and disruption of maternal-fetal immune tolerance; (C) A forest plot displaying the association (with 95% confidence intervals) between oral bacteria and adverse pregnancy outcomes (preterm birth, low birth weight, and gestational diabetes); this plot quantifies risk effect sizes and highlights the significant correlation between oral pathogens and these outcomes. NF-κB: Nuclear factor kappa-B signaling pathway .

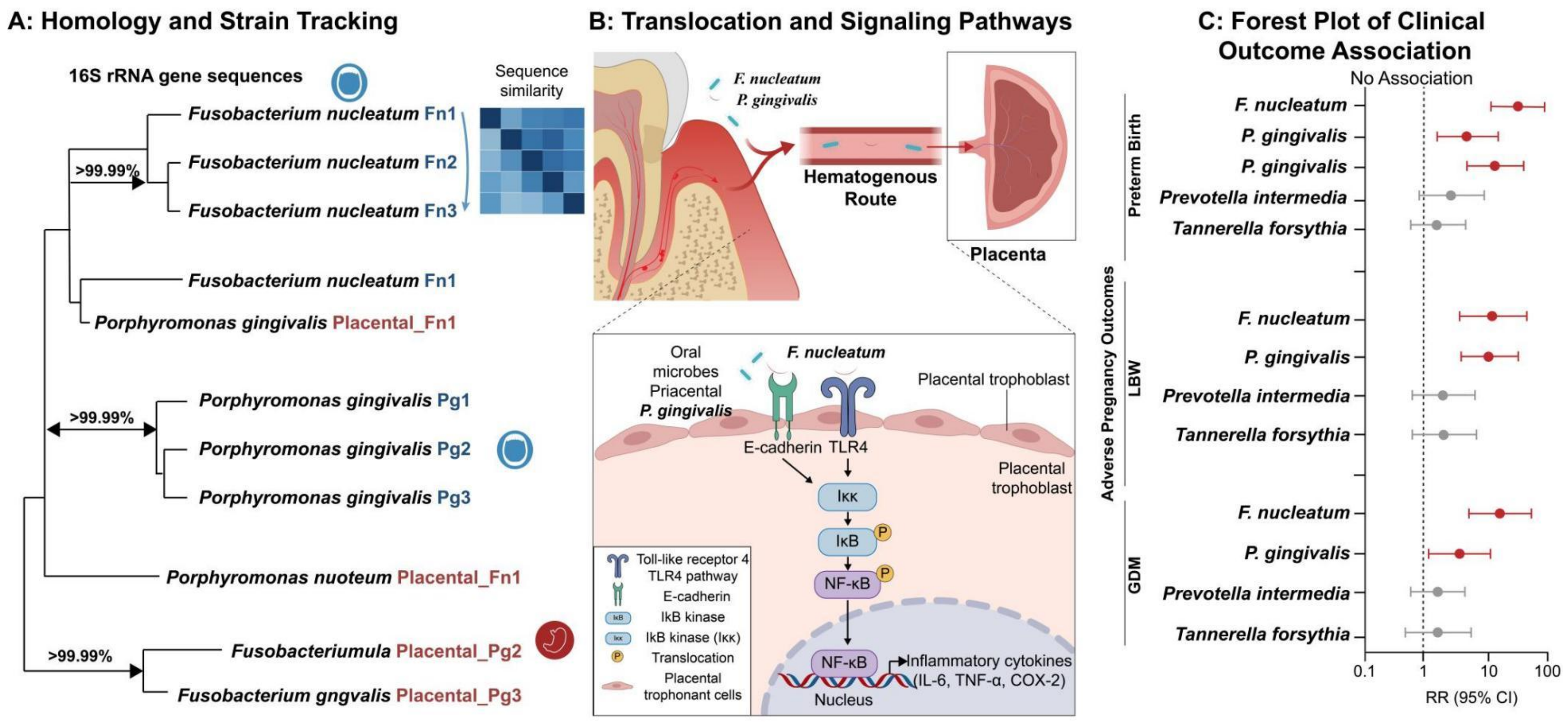

Figure S4: Annual Publication Volume, Keyword Burst, and Disciplinary Knowledge Flow in Oral-Placental Research from 2016 to 2025. (A) Annual publication volume and keyword burst analysis: the area chart shows a steady increase in annual publications (average annual growth rate of 5.03%), with line annotations indicating burst keywords at each stage (2016–2019: preterm birth, periodontal disease; 2019–2022: Fusobacterium nucleatum, inflammation; 2023–2025: preeclampsia, gestational diabetes), clearly presenting the temporal evolution of research foci; (B) Disciplinary knowledge flow diagram: nodes represent five core disciplines (obstetrics and gynecology, periodontology, microbiology, immunology, reproductive medicine), with edge thickness indicating the intensity of knowledge citation between disciplines, revealing microbiology and immunology as core cross-disciplinary hubs, and the most frequent bidirectional knowledge flow between stomatology and obstetrics and gynecology, reflecting the interdisciplinary integration characteristics of the field.

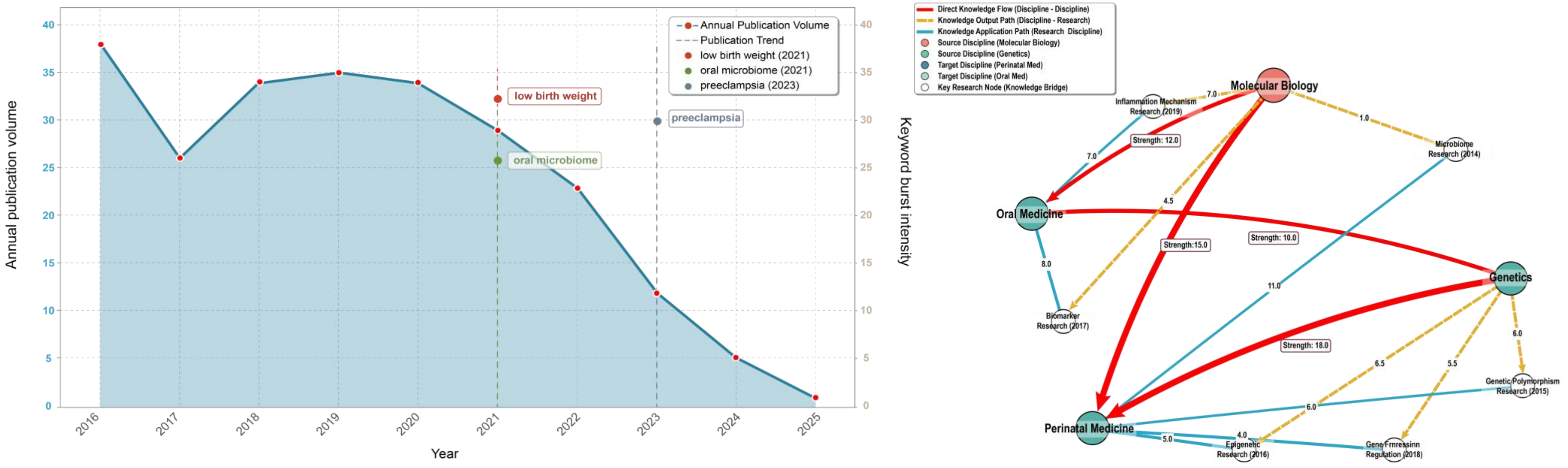

Figure S5. Potential Molecular Pathological Associations between Oral Health and Placental Function in the Oral-Placental Axis. This figure constructs a bidirectional regulation model of the oral-placental axis, integrating two core pathways: direct and indirect. The indirect pathway involves oral dysbiosis inducing systemic inflammation and metabolic disorders, remotely affecting the placenta via circulating factors without causing primary placental pathological damage; ② Direct pathway: oral pathogens invade the placenta through hematogenous transmission, sharing molecular mechanisms such as oxidative stress, immune dysregulation, and epithelial barrier damage; oral pathological markers can serve as early warning indicators of placental dysfunction; the two pathways converge on key signaling pathways such as NF-κB and PI3K-AKT, forming a systemic network of oral-placental associations, providing a theoretical framework for pre-pregnancy intervention and precise prevention and treatment during pregnancy. OPA: Oral–placental axis; NF-κB: Nuclear factor kappa B signaling pathway; PI3K-AKT: Phosphatidylinositol 3-kinase and protein kinase B signaling pathway; IL-1: Interleukin 1; IL-6: Interleukin 6; TNF-α: Tumor necrosis factor alpha.

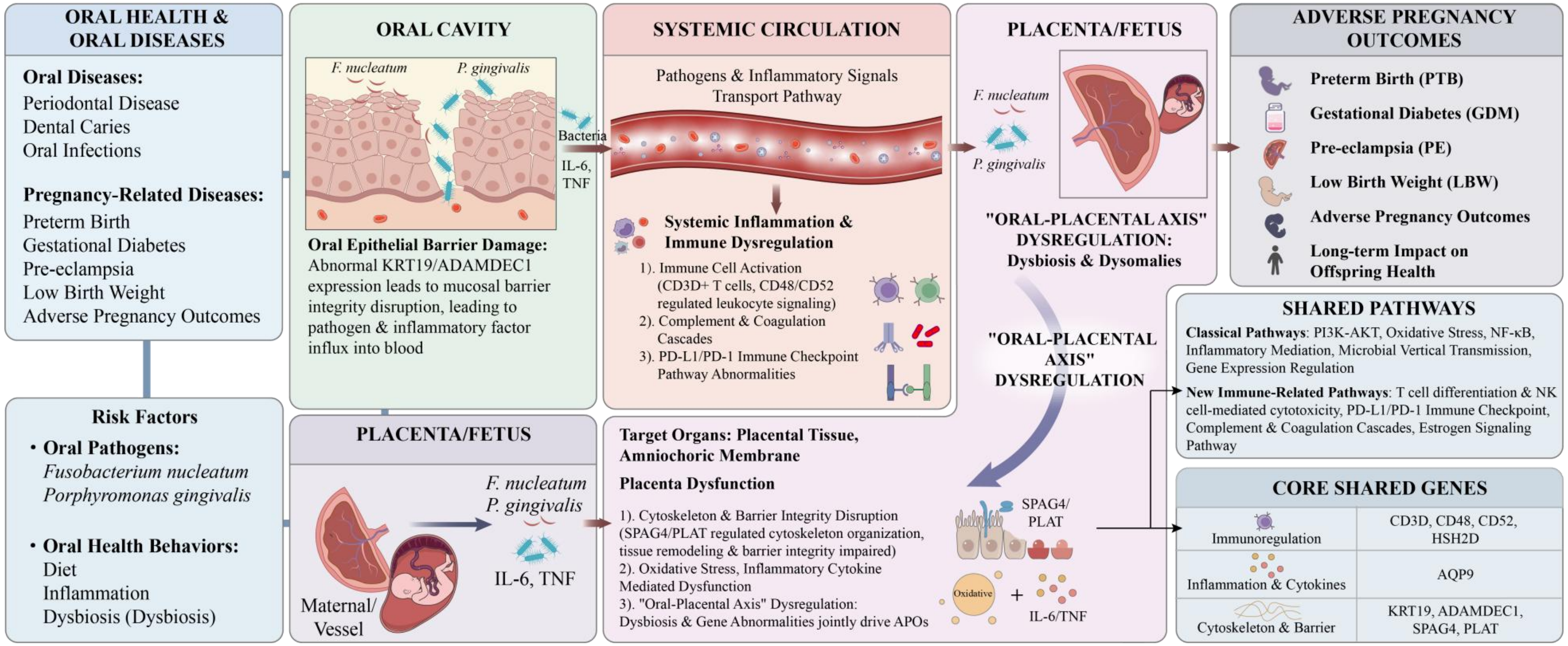

Supplementary Tables

Table S1: Search Strategy for Oral-Placental Axis Research (2016−2025). This table details the literature search protocols applied across three core biomedical databases (Web of Science Core Collection, Scopus, PubMed) for oral-placental axis research published between 2016 and 2025. It lists Boolean search terms, field restrictions (title/abstract/subject), exclusion criteria (animal studies, non-English, non-original/review papers), and initial record counts retrieved from each database. The standardized strategy ensures reproducibility and comprehensive coverage of eligible literature.

No.	Search Term	Citations
Web of Science	Web of Science	Web of Science
1	TS=(oral OR mouth OR dental OR stomatognathic OR buccal) AND TS=(placenta OR placental OR afterbirth OR "fetal membrane" OR "placental tissue") NOT TS=(animal OR animals OR mouse OR mice OR rat OR rats OR rabbit* OR dog OR dogs OR cat OR cats OR pig OR pigs OR swine OR primate* OR monkey* OR avian OR bird* OR zebrafish OR drosophila OR "fruit fly" OR "in vivo" OR veterinary)	1 AND 2 AND 3 AND 4/808
2	LA=ENGLISH	-
3	DT=(Article OR Review)	-
4	DOP=(2016-01-01/2025-12-31)	-
Scopus	Scopus	Scopus
1	TITLE-ABS-KEY (oral OR mouth OR dental OR stomatognathic OR buccal) AND TITLE-ABS-KEY (placenta OR placental OR afterbirth OR "fetal membrane" OR "placental tissue") AND NOT (animal OR animals OR mouse OR mice OR rat OR rats OR rabbit* OR dog OR dogs OR cat OR cats OR pig OR pigs OR swine OR primate* OR monkey* OR avian OR bird* OR zebrafish OR drosophila OR "fruit fly" OR "in vivo" OR veterinary) AND PUBYEAR > 2016 AND PUBYEAR < 2025 AND (LIMIT-TO (DOCTYPE,"ar") OR LIMIT-TO (DOCTYPE,"re")) AND (LIMIT-TO (LANGUAGE,"English"))	1080
PubMed	PubMed	PubMed
1	((("oral"[Title/Abstract] OR "mouth"[Title/Abstract] OR "dental"[Title/Abstract] OR "stomatognathic"[Title/Abstract] OR "buccal"[Title/Abstract]) AND ("placenta"[Title/Abstract] OR "placental"[Title/Abstract] OR "afterbirth"[Title/Abstract] OR "fetal membrane"[Title/Abstract] OR "placental tissue"[Title/Abstract])) NOT ("animal"[Title/Abstract] OR "animals"[Title/Abstract] OR "mouse"[Title/Abstract] OR "mice"[Title/Abstract] OR "rat"[Title/Abstract] OR "rats"[Title/Abstract] OR "rabbit*"[Title/Abstract] OR "dog"[Title/Abstract] OR "dogs"[Title/Abstract] OR "cat"[Title/Abstract] OR "cats"[Title/Abstract] OR "pig"[Title/Abstract] OR "pigs"[Title/Abstract] OR "swine"[Title/Abstract] OR "primate*"[Title/Abstract] OR "monkey*"[Title/Abstract] OR "avian"[Title/Abstract] OR "bird*"[Title/Abstract] OR "zebrafish"[Title/Abstract] OR "drosophila"[Title/Abstract] OR "fruit fly"[Title/Abstract] OR "in vivo"[Title/Abstract] OR "veterinary"[Title/Abstract])) AND ((classicalarticle[Filter] OR review[Filter]) AND (english[Filter]) AND (2016:2025[pdat]))	162

Table S2: Top 10 Most Productive Countries/Regions in Oral-Placental Axis Research (2016−2025). This table ranks the top 10 countries/regions by total publication output in oral-placental axis research, presenting key metrics including total publications, citation counts, single-country publications (SCP), multi-country publications (MCP), and international collaboration ratio (MCP Ratio). It reflects global research distribution, academic influence, and cross-border collaboration patterns in the field.

Rank	Country	Articles	Citations	SCP	MCP	MCP Ratio
1	USA	52	17305	16	36	69.2
2	CHINA	32	2778	17	15	46.9
3	AUSTRALIA	16	2737	2	14	87.5
4	ITALY	16	601	5	11	68.8
5	INDIA	14	551	7	7	50
6	UNITED KINGDOM	12	487	3	9	75
7	FINLAND	8	620	1	7	87.5
8	CANADA	7	1242	0	7	100
9	JAPAN	7	264	1	6	85.7
10	SPAIN	7	327	0	7	100

Table S3: Institutional Publication Output Ranking in Oral-Placental Axis Research (2016–2025). This table lists the top 10 research institutions ranked by publication count, with core indicators including total publications, citation counts, and total link strength (collaboration intensity). It identifies leading institutions and their academic influence, revealing key research hubs in the field.

id	Organization	Documents	Citations	Total link strength
1	Univ Queensland	7	413	8
2	Univ Melbourne	4	54	1
3	Univ Calgary	3	149	1
4	Univ Chile	3	55	7
5	Univ Los Andes	3	88	8
6	Univ Michigan	3	47	4
7	Brigham & Womens Hosp	2	25	18
8	Chu Besancon	2	42	17
9	Harvard Med Sch	2	25	18
10	Jazan Univ	2	31	2

Table S4. Detailed Profile of Highly Productive Authors in Oral-Placental Axis Research. This table provides comprehensive information for the most productive authors, including country, affiliation, publication count, citations, first/corresponding author proportions, research focus, publication timeline, collaboration network characteristics, and academic contributions. It highlights key scholars and their research strengths in the field.

Rank	Author Name	Country	Affiliation	Number of/Publications	Citation Count	First-Author Publications/Proportion	Corresponding-Author Publications/Proportion	Research Direction	Publication Time Series	Collaboration Network Characteristics	Research Background and Outcome Features
1	Balaji, Thodur Madapusi	India	Sri Ramachandra Institute of Higher Education and Research	3	33	2/66.7%	1/33.3%	Focusing on the correlation mechanism between oral microbiota and placental microecology, and exploring the pathogenic pathway of oral pathogenic bacteria on placental tissue	2019-2023 (1 publication per year on average)	Total connection strength: 12; moderate collaboration network with stable cooperation with mid-to-high productive authors in the field, mainly based on inter-institutional collaboration	A leading researcher in the interdisciplinary field of microbiology and reproductive medicine; research outcomes provide fundamental mechanistic evidence for adverse pregnancy outcomes caused by oral pathogenic bacteria, with an average of 11 citations per paper; outcomes focus on basic experimental research
2	Chaparro, Alejandra	Chile	Universidad de los Andes	3	88	2/66.7%	1/33.3%	Associative research on periodontal disease and adverse pregnancy outcomes, the impact of oral health status on placental function, and corresponding intervention strategies	2018-2024 (1 publication in 2018, 2021 and 2024 respectively)	Total connection strength: 14; a core node in the field's collaboration network with coverage across multiple institutions in Chile, Australia and other countries, and prominent characteristics of interdisciplinary collaboration (Oral Medicine + Perinatal Medicine)	A core scholar in the field of perinatal oral medicine in Chile; focusing on clinical epidemiological research, revealing the correlation rules between periodontitis and preterm birth, low birth weight in South America; research outcomes with high citation frequency and important clinical reference value

3	Gomez-Arango, Luisa F.	Australia	The University of Queensland	3	309	2/66.7 %	1/33.3 %	The impact of gestational diabetes mellitus complicated with oral disease on placental metabolism, and the vertical transmission mechanism of placental microbiome and oral microbiota	2020-2024 (1 publication in 2020, 2022 and 2024 respectively)	Total connection strength: 7; relatively loose collaboration network mainly based on intra-institutional cooperation, with Nitert, Marloes Dekker as the core collaborator	A key scholar in the field of perinatal medicine at The University of Queensland; dedicated to the interdisciplinary research of placental microecology and metabolic diseases, and analyzing the oral-placental microbial axis by multi-omics technologies; with an average of 103 citations per paper, outcomes published in high-impact journals and serving as benchmark research in the field
4	Hernandez, Marcela	Chile	Universidad de los Andes	3	55	1/33.3 %	2/66.7 %	Invasion mechanism of oral pathogenic bacteria (Fusobacterium nucleatum, etc.) on placental membranes, and screening of oral-derived risk factors for adverse pregnancy outcomes	2017-2022 (1 publication in 2017, 2020 and 2022 respectively)	Total connection strength: 10; moderate collaboration network with Chaparro, Alejandra as the core research team, mainly based on inter-institutional collaboration within Chile	An important scholar in the field of oral and perinatal research in Chile; focusing on the mechanistic research of pathogenic microorganisms and establishing a correlation model between oral pathogenic bacteria and placental inflammation; research outcomes provide experimental evidence for clinical risk factor screening
5	Ma, Guangyu	China	Stomatological Hospital, Air Force Medical University of the Chinese People's Liberation Army	3	8	1/33.3 %	1/33.3 % (1 co-authored paper without corresponding author)	Immune regulation mechanism of oral mucosal tissue and placental tissue, and the impact of oral immune microenvironment during pregnancy on placental immunity	2021-2025 (1 publication in 2021, 2023 and 2025 respectively)	Total connection strength: 8; loose collaboration network mainly based on small-scale Sino-Western cooperation with a small number of collaborators	Focusing on the interdisciplinary field of oral and placental immunity, with research focusing on basic immune mechanisms and outcomes published in mid-to-low impact journals; no highly cited outcomes yet, and identified as an explorer of emerging research directions in the field
6	Nitert, Marloes	Australia	The University	3	309	2/66.7 %	1/33.3 %	Correlation between placental epigenetic	2020-2024 (1 publication	Total connection strength: 7; relatively	A world-renowned scholar in the field of perinatal epigenetics;

	Dekker		of Queensland					modification and oral microbiota, and the placental-mediated mechanism of oral disease during pregnancy on fetal growth and development	in 2020, 2022 and 2024 respectively)	loose collaboration network with Gomez-Arango, Luisa F. as the core partner, mainly based on intra-laboratory collaboration within The University of Queensland	applying epigenetic technologies to oral-placental correlation research and for the first time revealing the regulatory effect of oral microbiota on placental DNA methylation; with an average of 103 citations per paper, outcomes laying a fundamental foundation for epigenetic research in the field
7	Rautava, Jaana	Finla nd	University of Turku	3	113	2/66.7 %	1/33.3 %	Maternal oral origin of neonatal microbiome, vertical transmission pathway of placental microbiome and oral microbiota, and perinatal influencing factors of children's oral health	2016-2020 (1 publication in 2016, 2018 and 2020 respectively)	Total connection strength: 11; moderate collaboration network, forming a core Finnish research team with Rautava, Samuli and Syrjanen, Stina	A core scholar in the field of perinatal microbiology in Finland; dedicated to the research on vertical transmission of maternal-neonatal microbiota and pioneering the exploration of the oral-placental-neonatal microbial transmission chain; research outcomes providing theoretical basis for perinatal intervention of children's oral health with citation frequency ranking among the top in the field
8	Rautava, Samuli	Finla nd	University of Turku	3	113	1/33.3 %	2/66.7 %	Highly synergistic research direction with Rautava, Jaana, focusing on the long-term impact of perinatal oral microbiota on the placenta and offspring, and the interaction between oral microbiome and placental immune microenvironment	2016-2020 (1 publication in 2016, 2018 and 2020 respectively)	Total connection strength: 11; moderate collaboration network, as a core member of the Finnish core research team with collaboration with internal institutions of the University of Turku and multiple institutions in European countries	A scholar in the interdisciplinary field of perinatal immunity and microbiology in Finland, proficient in combined analysis of microbiomics and immunomics; collaborative outcomes with Rautava, Jaana providing important evidence for maternal-neonatal microbial transmission in the field with highly prospective research
9	Rice, Gregory	Unsp ecifie	Unspecified	3	88	2/66.7 %	1/33.3 %	Molecular mechanism of periodontitis and placental	2019-2023 (1 publication	Total connection strength: 14; a core	An important international scholar in the field of molecular

		d						inflammation, oral-placental signaling pathway mediated by inflammatory factors, and oral-derived inflammatory intervention for adverse pregnancy outcomes	in 2019, 2021 and 2023 respectively)	node in the field's collaboration network with coverage across multiple European and American countries, and prominent characteristics of interdisciplinary collaboration (Oral Medicine + Molecular Biology + Perinatal Medicine)	mechanism research of oral and perinatal medicine; focusing on inflammatory signaling pathway research and revealing the role of inflammatory factors such as IL-6 and TNF-α in oral-placental signal transduction; outcomes providing molecular targets for anti-inflammatory intervention with high citation frequency
10	Syrjanen, Stina	Finla nd	University of Turku	3	113	1/33.3 %	2/66.7 %	Correlation between oral precancerous lesions and placental function (during pregnancy), the impact of oral microbial metabolites on placental nutrient transport, and the long-term maternal-neonatal impact of perinatal oral disease	2017-2021 (1 publication in 2017, 2019 and 2021 respectively)	Total connection strength: 11; moderate collaboration network, as a member of the Finnish core research team mainly based on cross-country and inter-institutional collaboration within Europe	A scholar in the interdisciplinary field of oral oncology and perinatal medicine in Finland; pioneering the exploration of the impact of oral precancerous lesions during pregnancy on the placenta and offspring with unique research directions; outcomes filling the research gap in the field and providing a new perspective for the comprehensive management of oral disease during pregnancy

Table S5: Detailed Profile of the Top 10 Most Cited Papers in Oral-Placental Axis Research. This table summarizes the top 10 highly cited publications, including publication year, research direction, core conclusions, journal, authors/affiliations, citation count, reasons for high citation, and academic contributions. It identifies landmark studies and pivotal findings shaping the field.

Rank	Paper Title	Year of Publication	Research Direction	Core Conclusion	Journal	First Author & Affiliation	Citation Count	Analysis of High Citation Reasons	Core Academic View/Fundamental Contribution
1	The placenta harbors a unique microbiome	2014	Maternal microbiota and fetal development	Maternal gut microbiota can regulate fetal immune system development	Science Translational Medicine	Aagaard K (Baylor College of Medicine and Texas Children's Hospital)	22	Proposing the concept of "intrauterine microbiota" and exploring its immune impact at an early stage with pioneering viewpoints	Challenging the traditional view of "sterile placenta" and putting forward a new hypothesis that maternal microbiota may affect the fetus through certain mechanisms
2	Human placenta has no microbiome but can contain potential pathogens	2019	Placental microbiome	Proving that the healthy term placenta has no inherent and abundant microbiome through strictly controlled research	Nature	De Goffau MC (University of Cambridge and Wellcome Sanger Institute)	14	Published in a top journal, clarifying an important controversy in the field with rigorous methods and achieving great influence of conclusions	Providing strong evidence for the "sterile placenta theory", emphasizing that previous studies may be affected by contamination and promoting the standardization of research methods
3	Microbiome of the placenta in pre-eclampsia supports the role of bacteria in the multifactorial cause of pre-eclampsia	2015	Pregnancy and vaginal microbiota	Exploring the changes in vaginal microbial community during pregnancy and its correlation with pregnancy outcomes	Journal of Obstetrics and Gynaecology Research	Amarasekara R (University of Cambridge and Wellcome Sanger Institute)	12	Studying the vaginal microbiota during pregnancy in a specific population (Sri Lanka) and providing regional clinical data	Enriching the understanding of the dynamic changes in vaginal microecology during pregnancy and suggesting its potential correlation with clinical problems such as preterm birth
4	Does the human placenta delivered at term have a microbiota? Results	2019	Childbirth and neonatal microbiota colonization	Reviewing how delivery mode shapes the initial microbial	American Journal of Obstetrics and	Theis KR (University of Michigan)	9	Systematically summarizing the key evidence of the impact of delivery	Clearly elaborating the fundamental differences between vaginal delivery and cesarean section in the

	of cultivation, quantitative real-time PCR, 16S rRNA gene sequencing, and metagenomics			community of neonates	Gynecology			mode on neonatal microbiota, as an important review	establishment of neonatal microbiota, guiding public health and clinical practice
5	The maternal microbiota drives early postnatal innate immune development	2016	Maternal microbiota and fetal immunity	Animal experiments proving that maternal gut microbial metabolites can cross the placenta and shape the offspring's immune system	Science	De Agüero MG (Centre Hospitalier Universitaire Vaudois (CHUV) and University of Lausanne)	9	Published in the top journal Science, for the first time proving the direct impact of maternal microbial metabolites on fetal immunity at the mechanistic level as a milestone study	Discovering the specific molecular mechanism (metabolites) by which maternal microbiota affects offspring immune development, pushing associative research to causal mechanistic research
6	Comparison of placenta samples with contamination controls does not provide evidence for a distinct placenta microbiota	2016	Placental microbiome	Detecting and describing the presence of a low-biomass unique microbial community in human placental tissue	Microbiome	Lauder AP (Temple University)	8	An important early study supporting the "placental microbiota theory", adopting advanced decontamination and analysis technologies	Reporting the compositional characteristics of the placental microbiome (non-pathogenic and similar to oral microbiota), supporting the hypothesis of oral-placental microbial transmission
7	Review: Maternal health and the placental microbiome	2017	Placental microbiome and preterm birth	Investigating the relationship between the presence of placental microbiota and preterm birth	Placenta	Pelzer E (Queensland University of Technology)	8	Directly exploring the potential link between placental microbiota and adverse pregnancy outcomes (preterm birth) with high clinical translational value	Combining placental microbiota research with clinical problems (preterm birth) and providing clues for finding predictive or intervention targets
8	A critical assessment of the "sterile womb" and "in utero colonization" hypotheses: implications for	2017	Intrauterine environment and microbiota	Critically reviewing the evidence and controversy of the existence of "intrauterine	Microbiome	Perez-Muñoz ME (Ottawa Research and Development Centre, Agriculture and	8	Sorting out the evidence of both sides at the peak of the controversy with a clear stand and guiding the thinking	Systematically evaluating the research quality, emphasizing the importance of strictly controlling contamination and playing a key role in standardizing

	research on the pioneer infant microbiome			microbiome"		Agri-Food Canada)		of the field	subsequent research
9	The preterm placental microbiome varies in association with excess maternal gestational weight gain	2015	Placental microbiome	Discovering the presence of non-pathogenic bacteria similar to oral microbiota in placental tissue by molecular technologies	American Journal of Obstetrics and Gynecology	Antony KM (Washington University in St. Louis)	7	An important follow-up study after the 2014 Aagaard study, further supporting the existence of a unique microbiota in the placenta	Strengthening the argument that "the placenta is not sterile", re-establishing the correlation between placental microbiota and maternal oral microbiota and supporting specific transmission pathways
10	Porphyromonas gingivalis within Placental Villous Mesenchyme and Umbilical Cord Stroma Is Associated with Adverse Pregnancy Outcome	2016	Dynamic changes of vaginal microbiota during pregnancy	Conducting a longitudinal study on the stability and changes of vaginal microbial community during pregnancy	PLOS ONE	Vanderpool SF (University of Louisville)	7	Providing detailed longitudinal data on vaginal microbiota changes during pregnancy with solid methods, as a basic research	Confirming the high stability of Lactobacillus-dominated vaginal microbiota in healthy pregnancy and setting a baseline for identifying abnormal changes

Table S6: Publication and Citation Metrics of Core Authors (Top 10). This table presents concise publication and citation data for the top 10 core authors, including publication count (top 10 papers), first-author publications, citations, H-index, country, and affiliation. It quantifies the academic impact of key contributors.

Rank	Author	Articles (of TOP10)	Published Articles (as First Author)	Citations	H-index	Country	Institution
1	TUOMINEN, HEIDI	3	3	113	3	FINLAND	UNIV TURKU
2	CHAPARRO, ALEJANDRA	2	2	60	2	CHILE	UNIV LOS ANDES
3	GOMEZ-ARANGO, LUISA F.	2	2	182	2	AUSTRALIA	UNIV QUEENSLAND;UNIV QUEENSLAND
4	JAJOO, NAMRATA S.	2	2	36	2	INDIA	DR HEDGEWAR SMRUTI RUGNA SEVA MANDALS DENT COLL AND
5	MAHENDRA, JAIDEEP	2	2	31	2	INDIA	MEENAKSHI AMMAL DENT COLL
6	YE, CHANGCHANG	2	2	84	2	JAPAN	TOKYO MED AND DENT UNIV
7	ABBADE, JOELCIO FRANCISCO	1	1	47	1	FINLAND	LUNENFELD-TANENBAUM RESEARCH INSTITUTE
8	ADLER, CHRISTINA JANE	1	1	18	1	AUSTRALIA	UNIV SYDNEY
9	AGRAWAL, RITU	1	1	0	0	INDIA	KD DENT COLL AND HOSP
10	AHMADIAN, ELHAM	1	1	39	1	IRAN	TABRIZ UNIV MED SCI

Table S7: Detailed Characteristics of the Top 20 Journals in Oral-Placental Axis Research. This table profiles the top 20 journals by publication volume, including publication count, journal partition, 2025 impact factor, research scope, average citations, journal orientation, and field adaptability. It identifies core publishing platforms and their academic positioning.

Rank	Journal Name	Number of/Publications	CAS Journal Partition/ (Major/Minor Discipline)	2025 Impact Factor	Research Topics of Included Literatures	Average Citation Count	Journal Orientation	Analysis of Field Adaptability
1	FRONTIERS IN MICROBIOLOGY	6	Biology (Zone 2)/Microbiology (Zone 3)	4.5	Whole field of microbiology (basic/medical/environmental/agricultural microbiology, virology, drug resistance, etc.)	Approx. 5.2	Open-access comprehensive journal in microbiology with a large publication volume and fast review speed	Adaptable to the research on microbiota-host interaction, oral microbiota and pregnancy-related microbiota
2	SCIENTIFIC REPORTS	6	Medicine (Zone 3)/Multidisciplinary (Zone 3)	3.9	Whole field of natural sciences (basic/clinical/interdisciplinary sciences)	Approx. 3.8	Comprehensive open-access journal with a wide acceptance scope and moderate acceptance threshold	Adaptable to the preliminary outcomes of interdisciplinary fields such as the oral-placental axis and perinatal medicine
3	PLACENTA	5	Medicine (Zone 3)/Obstetrics and Gynecology (Zone 2), Reproductive Biology (Zone 3), Developmental Biology (Zone 3)	2.5	Placental development, maternal-fetal interaction, pregnancy pathology, placental immunity/metabolism/molecular mechanism	Approx. 4.1	Authoritative specialized journal in placental research focusing on the combination of basic and clinical research	Highly adaptable to the core directions such as placental development, pre-eclampsia and maternal-fetal immunity
4	FRONTIERS IN ENDOCRINOLOGY	4	Medicine (Zone 3)/Endocrinology and Metabolism (Zone 3)	4.6	Whole field of endocrinology (reproductive endocrinology, pregnancy endocrinology, metabolic diseases, etc.)	Approx. 4.9	Open-access comprehensive journal in endocrinology with a large publication volume and short review cycle	Adaptable to the research on reproductive endocrinology, pregnancy metabolic disorders and placental endocrinology
5	JOURNAL OF MATERNA	4	Medicine (Zone 4)/Obstetrics and Gynecology	1.6	Maternal-fetal medicine, neonatal medicine, clinical and basic perinatal research	Approx. 2.3	Clinically oriented journal of perinatal medicine accepting clinical research and case reports	Adaptable to the research on maternal-fetal complications, neonatal outcomes and perinatal

	L-FETAL & NEONATA L MEDICINE		(Zone 4)					intervention
6	JOURNAL OF REPRODU CTIVE IMMUNOL OGY	4	Medicine (Zone 3)/Immunology (Zone 3), Reproductive Biology (Zone 3)	2.9	Whole field of reproductive immunology (pregnancy immunity, implantation immunity, reproductive infectious immunity, etc.)	Approx. 3.3	Specialized journal of reproductive immunology emphasizing both basic and clinical research	Highly adaptable to the research on maternal-fetal immune tolerance, immune mechanism of pre-eclampsia and reproductive infectious immunity
7	PERIODON TOLOGY 2000	4	Medicine (Zone 1, Top)/Dentistry and Oral Surgery (Zone 1)	15.7	Authoritative reviews of periodontology (periodontitis, periodontal therapy, correlation between periodontal disease and systemic diseases)	Approx. 16.8	Top review journal of periodontology publishing only invited high-quality reviews	Adaptable to the research on the mechanism of periodontitis and pregnancy complications as well as the oral-placental axis
8	REPRODU CTIVE SCIENCES	4	Medicine (Zone 3)/Obstetrics and Gynecology (Zone 4), Reproductive Biology (Zone 4)	2.5	Reproductive science (reproductive endocrinology, assisted reproduction, pregnancy diseases, reproductive molecular mechanism)	Approx. 2.6	Comprehensive journal of reproductive medicine focusing on basic and translational research	Adaptable to the research on reproductive endocrinology, assisted reproduction and the mechanism of pregnancy-related diseases
9	AMERICA N JOURNAL OF REPRODU CTIVE IMMUNOL OGY	3	Medicine (Zone 3)/Immunology (Zone 3), Reproductive Biology (Zone 3)	2.4	Reproductive immunology (pregnancy immunity, reproductive infection, immune infertility, reproductive tumor immunity)	Approx. 3.0	Established journal of reproductive immunology emphasizing the combination of basic and clinical research	Adaptable to the research on maternal-fetal immunity, pregnancy immune pathology and reproductive infectious immunity
10	CELLS	3	Biology (Zone 2)/Cell Biology (Zone 3)	5.2	Whole field of cell biology (cell signaling, stem cells, cell metabolism, cell immunity)	Approx. 6.8	Open-access comprehensive journal in cell biology with a large publication volume and wide acceptance scope	Adaptable to the research on placental cell biology, maternal-fetal cell interaction and oral cell mechanism
11	FRONTIER S IN MEDICINE	3	Medicine (Zone 3)/Medicine: Research and Experiment	3.0	Whole field of medicine (clinical/basic/translational medicine, obstetrics and gynecology, oral medicine, etc.)	Approx. 3.9	Open-access comprehensive medical journal with interdisciplinary acceptance and fast review speed	Adaptable to the research on the oral-placental axis, perinatal medicine and translational medicine

			(Zone 3)					
12	INTERNATIONAL JOURNAL OF MOLECULAR SCIENCES	3	Chemistry (Zone 2, Top)/Biochemistry and Molecular Biology (Zone 2)	4.9	Whole field of molecular sciences (molecular biology, molecular medicine, molecular immunology, molecular metabolism)	Approx. 7.9	Flagship open-access journal in molecular sciences with an extremely large publication volume and moderate acceptance threshold	Adaptable to the research on molecular mechanisms (placental molecules, oral molecules, pregnancy molecular pathways)
13	JOURNAL OF PERIODONTOLOGY	3	Medicine (Zone 2)/Dentistry and Oral Surgery (Zone 1)	3.8	Periodontology (periodontitis, periodontal regeneration, periodontal microbiota, clinical periodontal therapy)	Approx. 4.3	Authoritative journal of periodontology emphasizing both basic and clinical research	Adaptable to the research on periodontitis, oral microbiota and the correlation between periodontal disease and pregnancy
14	MICROBIOME	3	Biology (Zone 1, Top)/Microbiology (Zone 1)	12.7	Microbiomics (gut/oral/placental microbiome, microbiota-host interaction)	Approx. 15.2	Top journal of microbiomics focusing on microbiota and health/disease	Highly adaptable to the research on oral microbiome, placental microbiome and maternal-fetal microbiota
15	NUTRIENTS	3	Medicine (Zone 3)/Nutrition (Zone 3)	5.0	Nutrition (nutrition and pregnancy, nutrition and oral health, nutritional metabolism, nutritional intervention)	Approx. 5.3	Open-access comprehensive journal in nutrition with a large publication volume and wide acceptance scope	Adaptable to the research on pregnancy nutrition, oral nutrition and nutrition-placenta interaction
16	ORAL DISEASES	3	Medicine (Zone 3)/Dentistry and Oral Surgery (Zone 3)	2.9	Oral diseases (periodontal disease, oral infection, oral mucosal disease, correlation between oral and systemic diseases)	Approx. 3.9	Comprehensive journal of oral medicine emphasizing both basic and clinical research	Adaptable to the research on oral diseases and the correlation between oral and systemic (pregnancy) diseases
17	PLOS ONE	3	Medicine (Zone 3)/Multidisciplinary (Zone 3)	2.6	Whole field of natural sciences (basic/clinical/interdisciplinary sciences)	Approx. 3.5	Comprehensive open-access journal with a low acceptance threshold and large publication volume	Adaptable to the preliminary outcomes of interdisciplinary fields such as the oral-placental axis and perinatal medicine
18	ACTA OBSTETRICIA ET GYNECOLOGICA SCANDINAVICA	2	Medicine (Zone 3)/Obstetrics and Gynecology (Zone 3)	3.1	Obstetrics and Gynecology (pregnancy complications, clinical obstetrics, reproductive health, perinatal medicine)	Approx. 3.0	Authoritative Nordic journal of obstetrics and gynecology with clinical orientation	Adaptable to the research on clinical obstetrics, pregnancy complications and perinatal medicine

19	AMERICAN JOURNAL OF OBSTETRICS AND GYNECOLOGY	2	Medicine (Zone 1, Top)/Obstetrics and Gynecology (Zone 1)	8.4	Top journal of obstetrics and gynecology (maternal-fetal medicine, clinical obstetrics/gynecology, reproductive medicine)	Approx. 9.8	Top clinical journal of obstetrics and gynecology accepting high-quality clinical and basic research	Highly adaptable to the top research on maternal-fetal medicine, pre-eclampsia and pregnancy complications
20	BIOMOLECULES	2	Biology (Zone 2)/Biochemistry and Molecular Biology (Zone 2)	4.8	Biomolecules (nucleic acids, proteins, lipids, carbohydrates, correlation between biomolecules and diseases)	Approx. 5.7	Open-access comprehensive journal in biomolecular science with a wide acceptance scope	Adaptable to the research on placental biomolecules, oral biomolecules and pregnancy molecular mechanism

Table S8: Comprehensive Statistics of Core Keywords in Oral-Placental Axis Research. This table quantifies core research keywords with metrics including frequency, centrality, first occurrence year, burst strength, burst period, associated topics, and evolutionary significance. It reflects research hotspots and thematic shifts over time.

No.	Core Keyword	Frequency	Centrality	First Year of Appearance	Burst Strength	Burst Time Period	Keyword-Associated Topics	Remarks (Topic Evolution Correlation)
1	Preterm birth	34	0.13	2016	1.13	2018-2019	Periodontal disease and preterm birth, oral pathogenic bacteria-induced preterm birth, oral-derived risk factors for preterm birth	Basic core topic of the field, the most frequent keyword; forming the core of early research together with periodontal disease and associated with the core direction of adverse pregnancy outcomes
2	Periodontal diseases	28	0.14	2016	1.8	2018-2021	Periodontal disease and placental injury, periodontal disease and adverse pregnancy outcomes, prevention and treatment of periodontal disease	Basic core topic of the field; forming the dual core of early research together with preterm birth and promoting the formation of the "disease phenotype correlation" topic
3	Gestational diabetes mellitus	25	0.08	2017	1.7	2023-2025	Diabetes complicated with oral disease, placental metabolism, oral-placental metabolic axis	Subdivided core keyword of the field focusing on special populations and promoting the research direction of "precision population intervention"
4	Amniotic fluid	18	0.25	2016	1.02	2017-2018	Amniotic fluid microbiota, amniotic fluid and placental inflammation, amniotic fluid as a medium for oral-placental correlation	Key hub keyword of the field with the highest centrality, connecting various research topics and playing a significant bridging role
5	Bacteria	17	0.18	2016	1.5	2018-2020	Oral bacteria, placental bacteria, bacteria-mediated inflammatory response	Core direction of mechanistic and associative analysis, serving as a basic keyword supporting microbiota-related research
6	Association	17	0.14	2018	1.1	2019-2021	Correlation between oral disease and placental lesions, correlation between microbiota and pregnancy outcomes	Core logic of research analysis, serving as a basic keyword supporting early phenotype correlation research
7	Fusobacterium nucleatum	13	0.06	2017	1.3	2019-2022	Fusobacterium nucleatum invasion of the placenta, virulence factors, placental injury mechanism	Subdivided core pathogenic bacterium of the field and the core of precise analysis of a single pathogenic bacterium in the medium term

8	Disease	13	0.14	2016	1.0	2017-2019	Oral disease, pregnancy-related disease, cross-tissue correlation mechanism of diseases	Basic research dimension of the field, connecting the research on oral and pregnancy-related diseases
9	Adverse pregnancy outcomes	12	0.06	2019	1.5	2017-2020	Preterm birth, low birth weight, pre-eclampsia, the impact of oral-placental correlation on pregnancy outcomes	Core research objective of the field, promoting the evolution of research towards clinical application orientation
10	Risk	11	0.10	2016	1.2	2017-2019	Pregnancy risk of oral disease, risk of placental lesions, risk assessment of adverse pregnancy outcomes	Core dimension of research analysis, providing theoretical support for risk early warning and intervention
11	Pregnancy	10	0.11	2017	2.0	2017-2021	Oral health during pregnancy, hormone changes, oral health care during pregnancy	Basic background keyword of the field and one of the keywords with the longest burst duration without obvious post-fluctuation decline in popularity
12	Oral microbiome	10	0.07	2021	1.12	2023-2025	Oral-placental microbial correlation, microbial vertical transmission, oral microbiomic analysis	Current persistent hotspot and a core symbol of the transformation from "phenotype correlation" to "mechanism exploration"
13	Periodontal pathogens	9	0.02	2020	1.6	2020-2021	Types of periodontal pathogens, invasion mechanism, correlation with placental inflammation	Subdivided direction of pathogenic bacterium research, supplementing the research dimension of oral pathogenic bacteria
14	Low birth weight	8	0.04	2021	2.64	2021-2023	Oral-derived factors of low birth weight, placental function and low birth weight	Keyword with the highest burst strength in the field and the core research focus from 2021 to 2023, highlighting the core status of adverse pregnancy outcomes
15	Delivery	8	0.05	2016	0.93	2016-2019	Delivery mode and oral microbial transmission, delivery outcome and oral-placental correlation	Subdivided direction of pregnancy outcomes, supplementing the research dimension of adverse pregnancy outcomes
16	Porphyromonas gingivalis	6	0.00	2020	2.42	2020-2021	Porphyromonas gingivalis and placental inflammation, action of virulence factors	Pathogenic bacterium with high burst strength with a sharp rise in attention from 2020 to 2021, the core of oral pathogenic bacterium research
17	Pre-eclampsia	6	0.01	2023	2.35	2023-2025	Oral-derived risk factors for pre-eclampsia, abnormal placental function and pre-eclampsia	Emerging hotspot in the later period, expanding the research dimension of adverse pregnancy outcomes
18	Gut microbiota	6	0.04	2021	1.35	2021-2022	Gut-oral-placental microbial axis, microbial metabolic interaction	Expansion direction of microbiomic research, promoting the research on multi-site microbial correlation
19	Vaginal	6	0.06	2016	2.28	2016-2019	Interaction between vaginal and oral	Core of early microbial interaction research

	microbiome						microbiota, vaginal microbiota and placental health	with a long burst duration
20	Diversity	6	0.05	2016	2.34	2016-2017	Oral microbial diversity, placental microbial diversity, correlation between microbiota diversity and pregnancy outcomes	Basic research keyword of microbiomics and an early research hotspot
21	Inflammation	6	0.07	2016	1.2	2019-2022	Placental inflammation, oral inflammation, inflammatory factor-mediated mechanism	Core mechanistic keyword throughout the whole cycle, supporting the evolution of topics from "phenomenon" to "mechanism"

Table S9: List of Co-expressed Shared Genes in Oral and Placental Diseases. This table lists key shared differentially expressed genes in both oral and placental lesions, including gene symbol, ID, expression patterns in oral/placental diseases, correlation strength, and core biological functions. It reveals conserved molecular mechanisms linking oral and placental pathology.

Gene	Gene ID	Expression in Oral Lesions	Expression in Placental Lesions	Oral-Placental Correlation	Core Function and Associated Mechanism
SPAG4	6676	High expression in OSCC	Abnormal expression in pre-eclampsia	Moderate	Cell proliferation, ciliary function; involved in tissue stress and homeostasis maintenance
HSH2D	54441	Insufficient data	Expression in infected/pathological placenta	Low	T cell immune adaptor protein; regulating local mucosal immunity
AQP9	366	Up-regulated in OSCC, promoting metastasis	Down-regulated in pre-eclampsia	High	Aquaglyceroporin; involved in metabolic reprogramming, immune migration and energy transport
KRT19	3880	Classic marker of oral cancer	Abnormal expression in trophoblasts	High	Epithelial cytoskeleton; involved in tissue damage repair and epithelial barrier function
CD52	1043	Expression in lymphocytes of oral immune diseases	High expression in trophoblasts & immune cells	Moderate	Immune regulation; affecting oral inflammation and maternal-fetal immune tolerance
PLAT	5327	Elevated in periodontitis	Abnormal expression in pregnancy vascular diseases	High	Fibrinolytic system, matrix degradation, vascular remodeling and inflammatory regulation
CD48	962	Expression in immune cells of oral inflammation	Expression in placental NK/T cells	Moderate	Immune costimulation; regulating mucosal and maternal-fetal interface immune crosstalk
CD3D	915	T cell marker	Placenta-infiltrating T cells	Moderate	Core component of T cell receptor; mediating adaptive immunity
ADAMDEC1	27299	High expression in the oral cancer microenvironment	Up-regulated in the placenta during pregnancy	Moderate	Matrix remodeling, inflammatory regulation; affecting the tissue microenvironment

Table S10: Co-occurring Bacterial Species in Oral and Placental Microbiota
This table summarizes bacterial species detected in both oral and placental microbiota, including detection sites, association with adverse pregnancy outcomes, sample size, detection rates, transmission notes, and references. It provides evidence for microbial transmission pathways.

No.	Bacterial Species (Latin/Common Name)	Detection Site/ (Oral/Placenta)	Correlation with Adverse Pregnancy Outcomes	Sample Size of Related Research (cases)	Oral Detection Rate	Placental Detection Rate	Notes on Vertical Transmission	Associated Literature Reference
1	Porphyromonas gingivalis	oral cavity, placenta	highly associated (preterm birth, preeclampsia)	132-600	30-60% (patients with periodontitis)	5.68%-51%	Distinct hematogenous dissemination, localized to the villous stroma	10.1371/journal.pone.0146157
2	Fusobacterium nucleatum	oral cavity, placenta	strongly associated (preterm birth, chorioamnionitis)	300-600	40-70%	15-35% (significantly increased in the preterm group)	Crosses the placental barrier via the hematogenous route	10.1016/j.anaerobe.2018.01.008
3	Streptococcus spp. (for example: S. mitis)	oral cavity, placenta	moderately associated (preterm birth, sepsis)	12-60	80-95%	10-44%	High similarity between oral and placental microbiota, of prenatal origin	10.1186/s12879-022-07530-z
4	Prevotella intermedia	oral cavity, placenta	moderately associated (preeclampsia)	50-200	20-50%	8-20%	Enters the systemic circulation via gingival bleeding	10.1111/jicd.12265
5	Ureaplasma urealyticum	oral cavity, placenta	highly associated (preterm birth, premature rupture of membranes)	20-130	-	15-30% (high in the preterm group)	May serve as a dual source of oral or ascending vaginal infection	10.3389/fcimb.2024.1486409
6	Capnocytophaga spp.	oral cavity, placenta	moderately associated (preterm birth independent of infection)	152	40-60%	-	The detection load is positively correlated with preterm birth	10.3389/fmed.2023.1177990
7	Treponema	oral cavity, placenta	moderately to highly	64	30-50%	The detection	Red complex bacteria,	10.1007/s00784-020-03287-4

	denticola		associated (threatened preterm birth)			frequency was significantly increased	which synergistically invade the placenta	
8	Lactobacillus spp.	oral cavity, placenta	negatively associated (exerts a protective effect on neurodevelopment)	807	60-90%	5-15%	Key commensal bacteria for maintaining placental microecological balance	10.1038/s41372-019-0505-8
9	Veillonella spp.	oral cavity, placenta	moderately associated (preterm birth)	152	60-90%	-	Low abundance in term pregnancy	10.3389/fmed.2023.1177990
10	Campylobacter rectus	oral cavity, placenta	moderately associated (preterm birth, preeclampsia)	-	15-40%	5-15%	Hematogenous spread to the placenta induces inflammation	10.2147/IJWH.S142730

Table S11: International Collaboration Data by Country/Region. This table details cross-country collaboration metrics, including publication count, core partners, total link strength, network role, and collaboration features. It maps global collaboration structure and regional research strengths.

Rank	Country/ Region	Number of/Publi cations	Core Collaboration Partners/ (Ranked by Collaboration Frequency)	Total Connectio n Strength	Role in Collaboratio n Network	Collaboration Feature Description
1	USA	53	Australia, China, UK, Canada, Finland	41	Core leading type	Global research core with the first place in publication volume, citation count (3440 times) and total connection strength; the highest international influence and collaboration activity, serving as the core hub of the national collaboration network; focusing on core research directions such as oral microbiota and placental microecology, and inflammatory mechanisms
2	China	35	USA, Australia, Japan, South Korea	11	Core leading type	Asian research core ranking second in publication volume with 1111 citations; international collaboration mainly based on China-US and China-Australia cooperation, focusing on clinical application and population research; serving as the core of the Asian regional collaboration network and actively participating in global core collaborative research
3	Australi a	17	USA, Finland, China, UK	20	Core leading type	One of the global research cores with the same publication volume as Italy (3rd place) and 919 citations; the total connection strength ranking among the top, forming a core collaboration triangle with the USA and Finland; focusing on directions such as oral pathogenic bacteria and placental injury, and the correlation with adverse pregnancy outcomes
4	Italy	17	USA, UK, France, Spain	11	Important participating type	An important European research node with the same publication volume as Australia (3rd place) and 322 citations; the collaboration network radiating across Europe and North America, focusing on subdivided directions such as the correlation between periodontal disease and pregnancy outcomes
5	India	16	USA, China, Singapore	15	Important participating type	An important Asian research country with 324 citations and relatively high total connection strength; international collaboration mainly based on US-India and China-India cooperation, focusing on clinical research and oral microbial detection; serving as an important supplement to the Asian regional collaboration network
6	Canada	10	USA, UK, Australia	13	Important participating type	An important North American research country with 406 citations; collaboration closely centering on core North American and Oceanian countries, focusing on basic research such as inflammatory factor-mediated mechanisms; serving as a supplementary node of the North American collaboration network

7	UK	9	USA, Australia, Canada, France	18	Important participating type	One of the European research cores with 177 citations and relatively high total connection strength; the collaboration scope covering Europe, America and Oceania, focusing on placental microecology and oral health care during pregnancy; serving as an important node of the European regional collaboration network
8	Finland	8	Australia, USA, Sweden, Norway	14	Important participating type	High international collaboration activity with 206 citations and close cooperative relations with Australia and the USA; focusing on oral pathogenic bacteria and placental inflammatory mechanisms, providing a large amount of reliable experimental data for the field
9	Japan	7	China, USA, South Korea	3	Peripheral participating type	An important Asian research country with 180 citations and low total connection strength; collaboration mainly based on China-Japan and US-Japan cooperation, focusing on oral microbiota and placental tissue repair; serving as a supplementary node of the Asian regional collaboration network
10	Spain	7	UK, USA, France	11	Peripheral participating type	An important European research country with 282 citations; collaboration centering on core European and North American countries, focusing on periodontitis and adverse pregnancy outcomes; serving as a supplementary node of the European regional collaboration network
11	France	6	UK, Italy, Spain	7	Peripheral participating type	A supplementary node of European research with 78 citations; collaboration concentrated within Europe, focusing on the correlation between oral disease and pregnancy-related diseases; providing subdivided direction support for the European collaboration network
12	Norway	6	Finland, Sweden, USA	8	Peripheral participating type	An important Nordic research node with 176 citations; collaboration closely centering on core Nordic and North American countries, focusing on placental microbial colonization research; supplementing Nordic regional research data
13	Turkey	6	USA, several European countries	3	Peripheral participating type	A West Asian research node with 23 citations; collaboration mainly with the USA, focusing on clinical basic research; filling the research gap in West Asian regions
14	Austria	5	Germany, Switzerland, several European countries	4	Peripheral participating type	A niche European research node with 33 citations; collaboration concentrated in Central Europe, focusing on basic oral microbial research; supplementing subdivided data for the European collaboration network
15	Chile	5	USA, Australia, Argentina	7	Peripheral participating type	South American research core with 104 citations; collaboration centering on core North American and Oceanian countries, focusing on oral-placental correlation research in regional populations; supplementing South American regional research evidence

Table S12: Research Focus of Core Institutions. This table outlines research priorities, core teams, representative outputs, collaboration networks, and research orientations of leading institutions. It clarifies institutional contributions and research paradigms.

No.	Core Institution/ (Country/Region)	Research Focus	Core Team Authors (Research Direction)	Representative Outcomes (Past 5 Years)	Collaboration Network/ (Core Collaborating Institutions + Collaboration Direction)	Research Orientation
1	The University of Queensland (Australia)	Correlation between oral microbiome and systemic diseases (e.g., preterm birth, cardiovascular disease); the impact of periodontal disease as chronic inflammation on pregnancy outcomes	Saso Ivanovski (Tissue Engineering, Peri-implantitis), Liza Brown (Periodontal Disease and Systemic Health)	Exploring the molecular mechanism of periodontal pathogens and placental inflammation, adverse pregnancy outcomes; developing novel therapies targeting oral pathogens	The University of Melbourne (Epidemiological Investigation), domestic hospitals (Clinical Sample Collection)	Translational research: Focusing on revealing the impact of oral disease on distant organs (including the placenta) from the perspective of microbiology and immunology, and exploring clinical intervention strategies
2	The University of Melbourne (Australia)	Oral epidemiology and public health; periodontal disease as a modifiable risk factor and its correlation with pregnancy complications such as gestational diabetes mellitus and preterm birth	Eric Reynolds (Oral Microbiota and Dental Caries/Periodontal Disease), Ajith Amarasinghe (Oral Public Health)	Data analysis of large cohort studies to evaluate the effectiveness of oral care intervention during pregnancy in improving maternal and infant health outcomes	The University of Queensland (Mechanistic Research), local health departments (Public Health Projects)	Population and clinical research: Focusing on verifying the correlation between oral health and pregnancy health through large-scale clinical data and public health projects, and promoting preventive medical policies
3	University of Calgary (Canada)	Maternal immune adaptation, inflammation and fetal programming during pregnancy; exploring how maternal infection (including potential oral infection) affects placental development and fetal neurodevelopment	Deborah Sloboda (Developmental Programming, Metabolic Health), James Reynolds (Perinatal Neuroscience)	Exploring the long-term impact of maternal systemic inflammation (possibly derived from periodontal disease) on placental epigenetics and fetal brain development	Collaboration with other Canadian universities (e.g., UBC, University of Alberta) in the field of developmental biology and neuroscience	Basic mechanistic research: In-depth exploration of the physiological and molecular changes of the maternal-placental-fetal axis under inflammatory stress, focusing on the developmental origins of health and disease theory

4	University of Chile (Chile)	Research on oral health and pregnancy outcomes in Latin American populations; the role of periodontal disease in pregnancy-induced hypertension	Andrea Maturana (Periodontal Disease and Medicine), Jorge Gamonal (Periodontology)	Case-control studies on specific populations to analyze the correlation between periodontitis and pregnancy-specific diseases such as pre-eclampsia	Latin American research networks with other universities, government health institutions	Regional clinical research: Focusing on local populations, studying the geographically characteristic oral disease burden and its impact on pregnancy complications
5	Universidad de los Andes (Chile)	Interdisciplinary research on oral medicine and systemic health; application of biomaterials and tissue engineering in oral and potential regenerative medicine	Few authors specialized in the oral-placental interdisciplinary field; the institution's strength lies in engineering and basic medicine	Potentially involving the study of host response to infection using tissue engineering models, but no prominent direct placental-associated research outcomes	Close collaboration with international engineering and materials science teams	Interdisciplinary basic research: Strength in bioengineering and basic science, but not a traditional core in specific clinical or translational research of the "oral-placental" axis
6	University of Michigan (USA)	Immune mechanism of periodontal disease onset; the oral cavity as a source of systemic inflammation and its contribution to chronic diseases (including adverse pregnancy outcomes)	William Giannobile (Periodontal Regeneration, Host-Directed Therapy), Dana Graves (Periodontal Immunology and Bone Immunology)	Studying how periodontal pathogens trigger the activation of specific immune cells (e.g., neutrophils, macrophages), thereby affecting the systemic inflammatory state	Harvard Medical School, other top US medical schools (collaboration in immunology and translational medicine)	Immune mechanism and translational frontier: Committed to clarifying the systemic impact of periodontitis from the fundamental immunological mechanism and developing novel immunomodulatory therapeutic strategies
7	Brigham & Women's Hospital (USA)	Obstetrics and Gynecology and Perinatal Medicine; prediction, prevention and mechanistic research of pregnancy complications (e.g., preterm birth, pre-eclampsia), focusing on maternal infection/inflammatory status	Thomas McElrath (Biomarkers of Preterm Birth), Ellen Seely (Pregnancy Metabolism and Endocrinology)	Identifying early pregnancy plasma biomarkers associated with maternal inflammation (including potential oral-derived inflammation)	Harvard Medical School (core teaching affiliated hospital with in-depth integration), Broad Institute (genomic analysis)	Top clinical translational research: At the forefront of perinatal clinical research, committed to translating basic discoveries (including inflammatory mechanisms) into bedside-available predictive tools and therapies
8	Chu Besancon (France)	A French regional medical center with practices in clinical periodontal therapy and oral health management during	Mainly clinical experts with relatively few large-scale mechanistic studies published internationally	Participating in or leading European multicenter clinical studies to evaluate the safety of periodontal therapy during pregnancy	French university hospital network, European Federation of Periodontology	Clinical practice and multicenter trials: Focusing on participating in and implementing international clinical guidelines, and

		pregnancy		and its improvement effect on pregnancy outcomes		conducting high-quality clinical efficacy verification within the European framework
9	Harvard Medical School (USA)	Systems biology and medicine; regarding the oral cavity as a microecosystem and studying its dynamic interaction with various systemic organs (including the placenta) in health				
